# Supplementary material for: Coordinated Ras and Rac Activity Shapes Macropinocytic Cups and Enables Phagocytosis of Geometrically Diverse Bacteria
Source: Curr Biol. 2020 Aug 3;30(15):2912–2926.e5. doi: 10.1016/j.cub.2020.05.049 (PMC7416115; doi:10.1016/j.cub.2020.05.049)
Supplement: Document S1. Figures S1–S5 [file mmc1.pdf]

Current Biology, Volume *30*

Supplemental Information

Coordinated Ras and Rac Activity Shapes Macropinocytic Cups and Enables Phagocytosis of Geometrically Diverse Bacteria

Catherine M. Buckley, Henderikus Pots, Aurelie Gueho, James H. Vines, Christopher J. Munn, Ben A. Phillips, Bernd Gilsbach, David Traynor, Anton Nikolaev, Thierry Soldati, Andrew J. Parnell, Arjan Kortholt, and Jason S. King

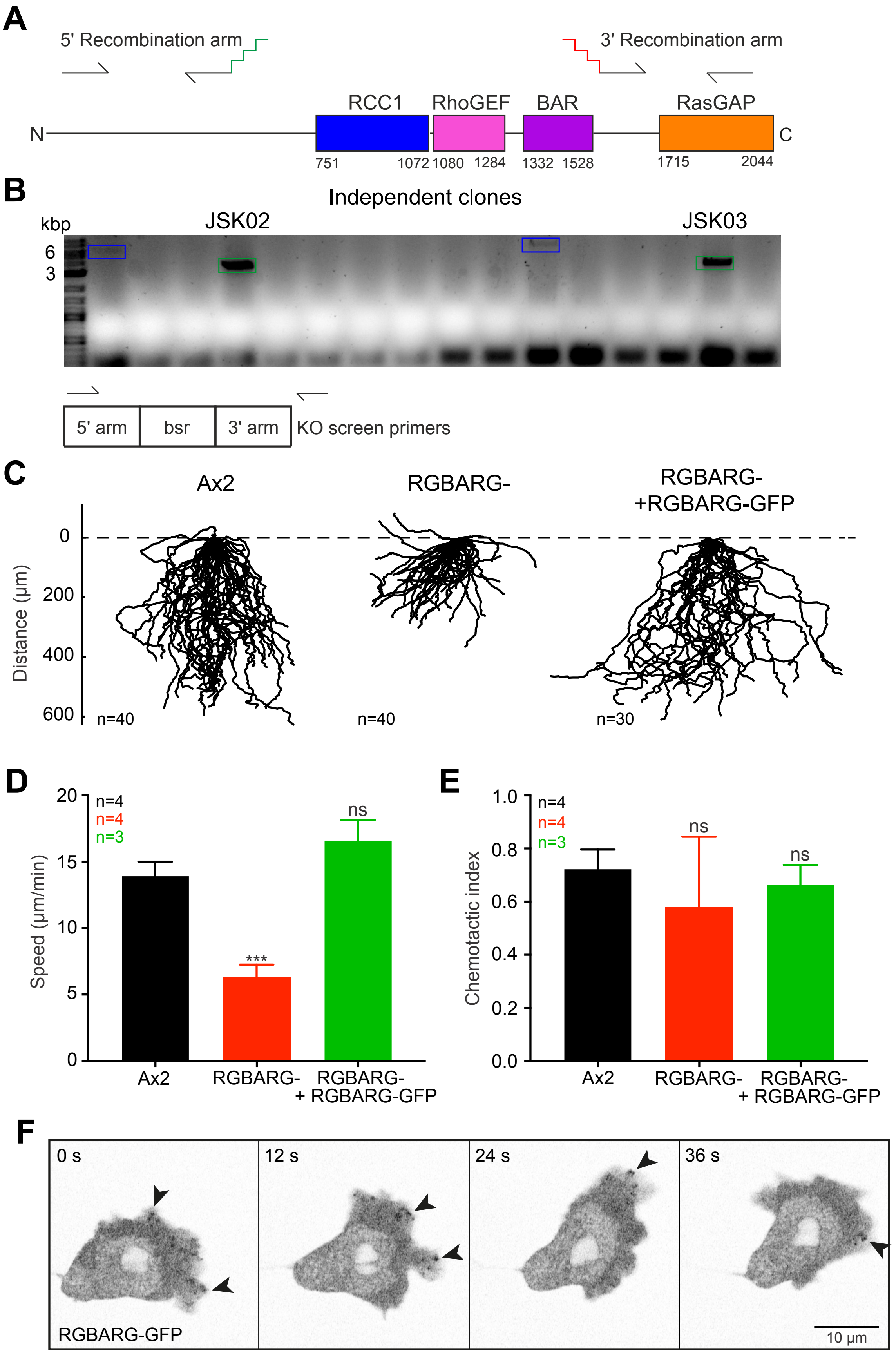

Figure S1: Generation of RGBARG mutants and analysis of chemotaxis, related to Figure 2. (A) Schematic of the *rgbA* genomic locus indicating the position of the regions encoding each domain, and the 5' and 3' recombination arms amplified by PCR. These were attached either side of a blasticidin selection cassette by fusion PCR and used to transform Ax2 cells and delete 3.6 kbp of the gene. (B) PCR screen of transformants, using one primer within the 5'recombination arm and another after the 3' arm. Clones with *rgbA* disrupted will give a product of 3.1 kbp (green box), the wild-type locus is 6.1 kbp (blue boxes). (C) Loss of RGBARG also disrupts chemotaxis. Tracks of Ax2, RGBARG- and RGBARG- cells rescued with RGBARG-GFP chemotaxing towards folate under agar for 45 minute movies. (D) Average cell speed and (E) Chemotactic index (movement up gradient/total pathlength). Bars indicate mean ± SEM. *** P<0.005 Mann-Whitney T-Test. (F) Localisation of RGBARG-GFP in Ax2 cells chemotaxing under agar. Arrows indicate the small, highly transient puncta observed.

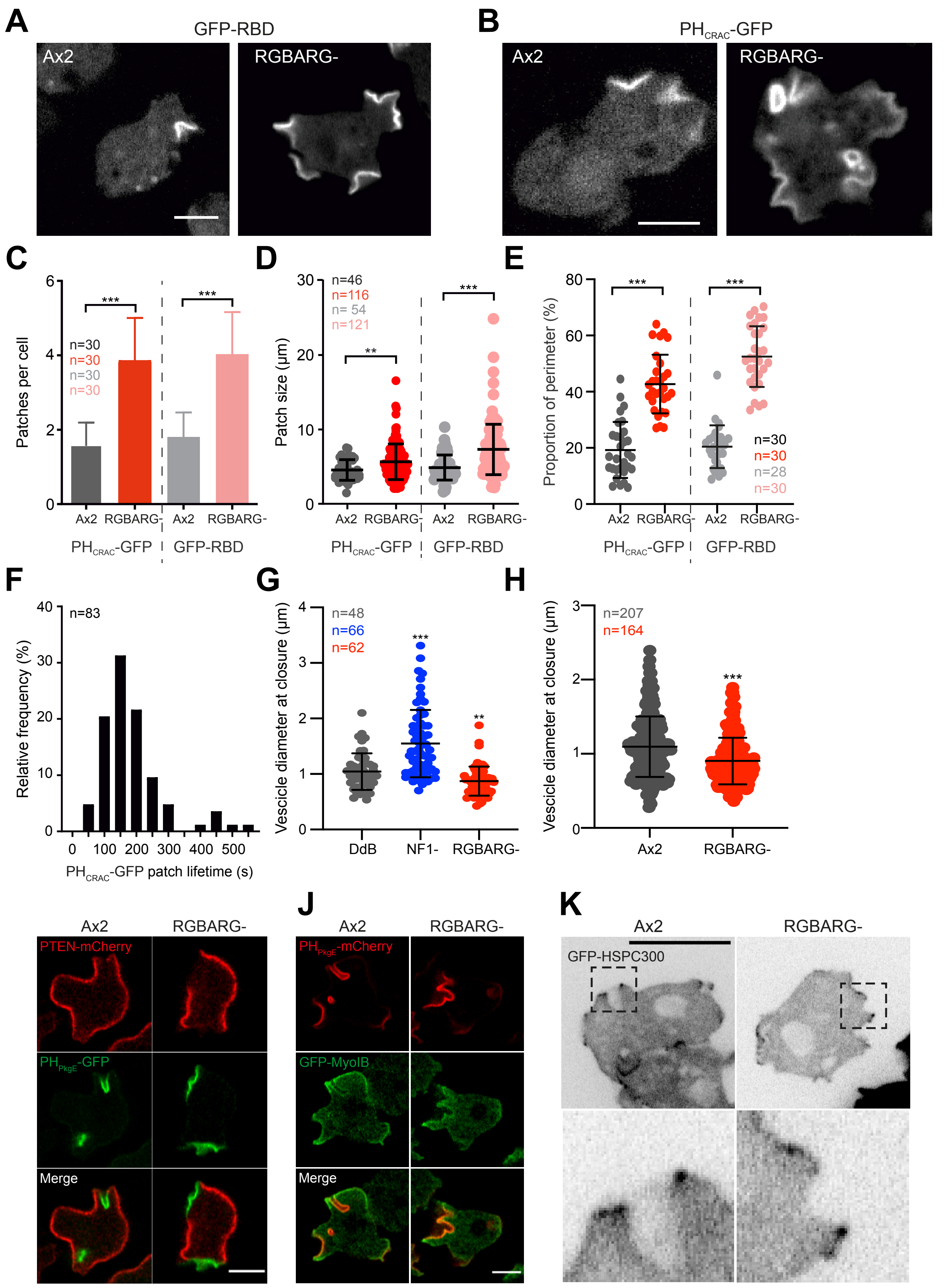

Figure S2: Analysis of cup signalling in Ax2-derived RGBARG mutants, related to Figure 3. (A) Single confocal planes of the active Ras probe GFP-RBD in Ax2 and RGBARG- cells. (B) Equivalent images using PH_{CRAC}-GFP. (C-E) Is quantification of the patch frequency, size and proportion of cell diameter respectively. (F) Histogram of PH_{CRAC}-GFP patch lifetime in Ax2 cells from maximum intensity projection movies (E.g. Movie 6). Lifetime was measured from the first frame an independent patch was visible to when it was completely removed from both the surface and any internalized vesicle. (G) and (H) is quantification of the size of the vesicles formed, as measured from 3D-projection timelapses of cells expressing (G) GFP-RBD or (H) PH_{CRAC}-GFP (Arrows on Figure 3 and see Movies 5 and 6). (I) Localisation of PTEN-mCherry and (J) MyoIB-GFP in Ax2 and RGBARG- cells, relative to forming cups, marked by the PIP_3 probe PHPkgE-GFP. (K) Recruitment of the HSPC300 subunit of the SCAR/WAVE complex to cup protrusions. Bottom panels are enlargements of the boxed regions above. All scale bars = 5 µm.

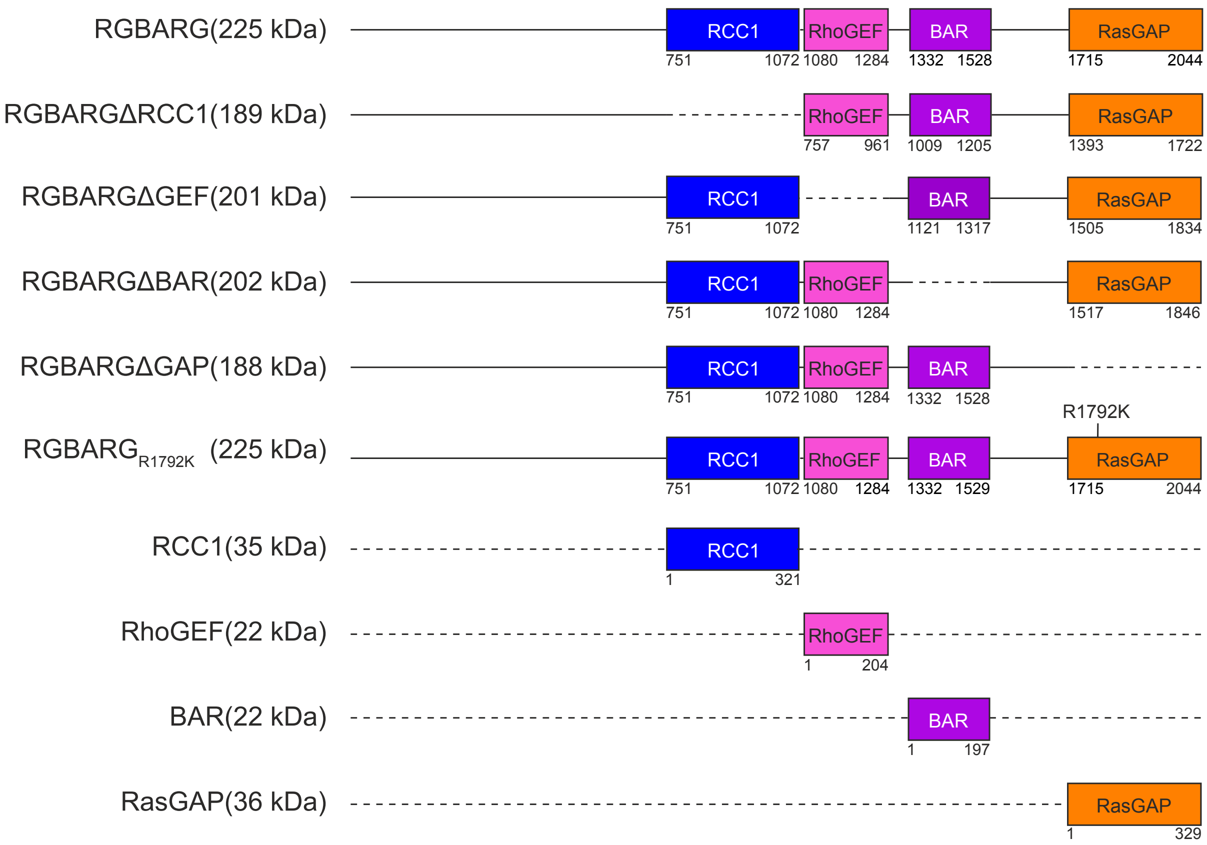

Figure S3: Schematic of the RGBARG truncation and point mutants used in this study, related to Figures 4 and 5

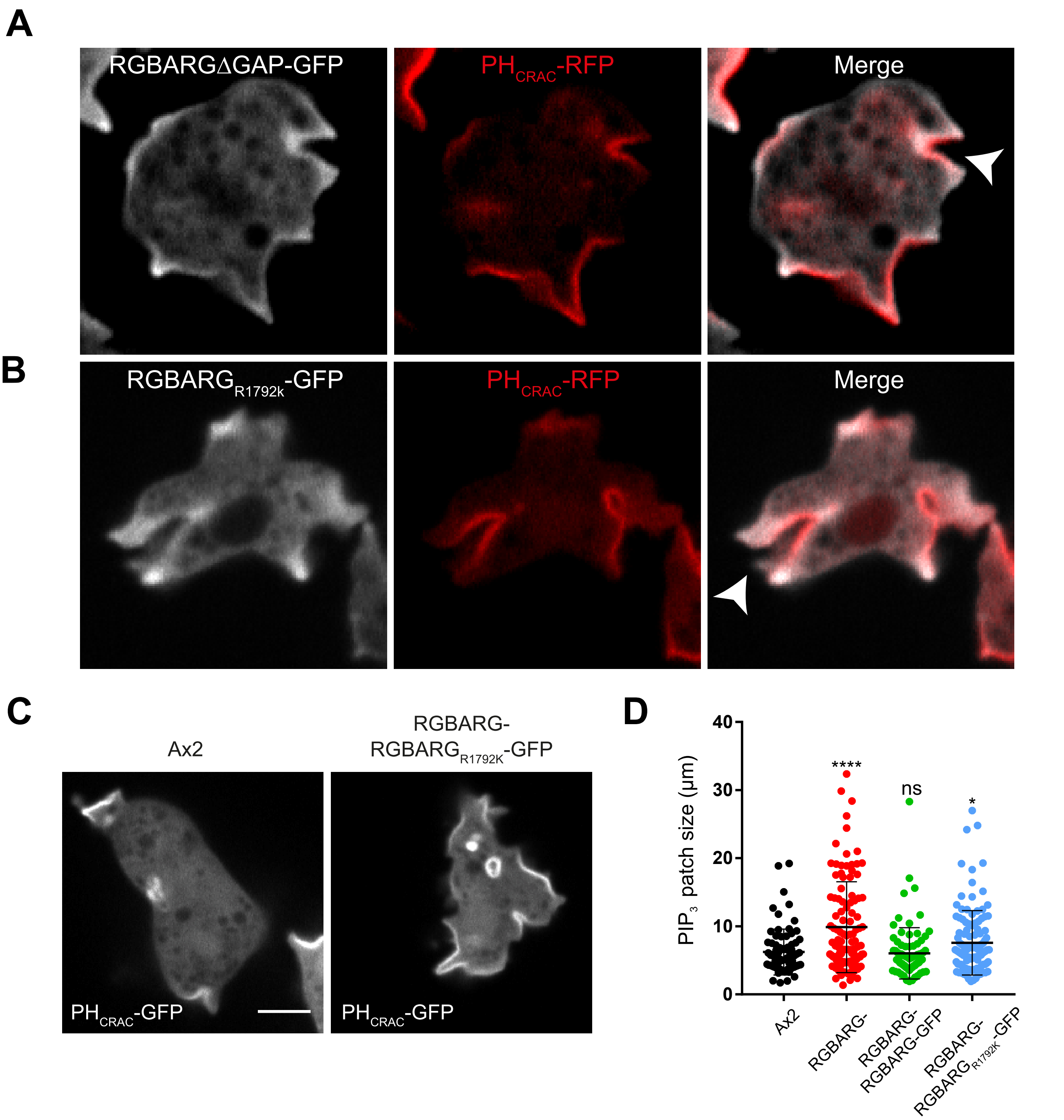

Figure S4: RGBARG$_{R1792K}$-GFP rescues RGBARG localisation, but not PIP$_3$ signalling, related to Figure 4. (A) Co-expression of RGBARGΔGAP-GFP and PH$_{CRAC}$-RFP in (Ax2) RGBARG- cells indicating that the RasGAP domain helps excluded RGBARG-GFP from PIP$_3$ rich regions of the cell. (B) The inactive GAP mutant R1792K localizes normally and is excluded from the base of protruding cups (arrowhead). (C) PH$_{CRAC}$-GFP localisation in Ax2 and RGBARG- cells expressing RGBARG$_{R1792K}$-GFP, demonstrating that PIP$_3$ dynamics are not rescued by this construct. (D) Quantification of PH$_{CRAC}$-GFP patch size.

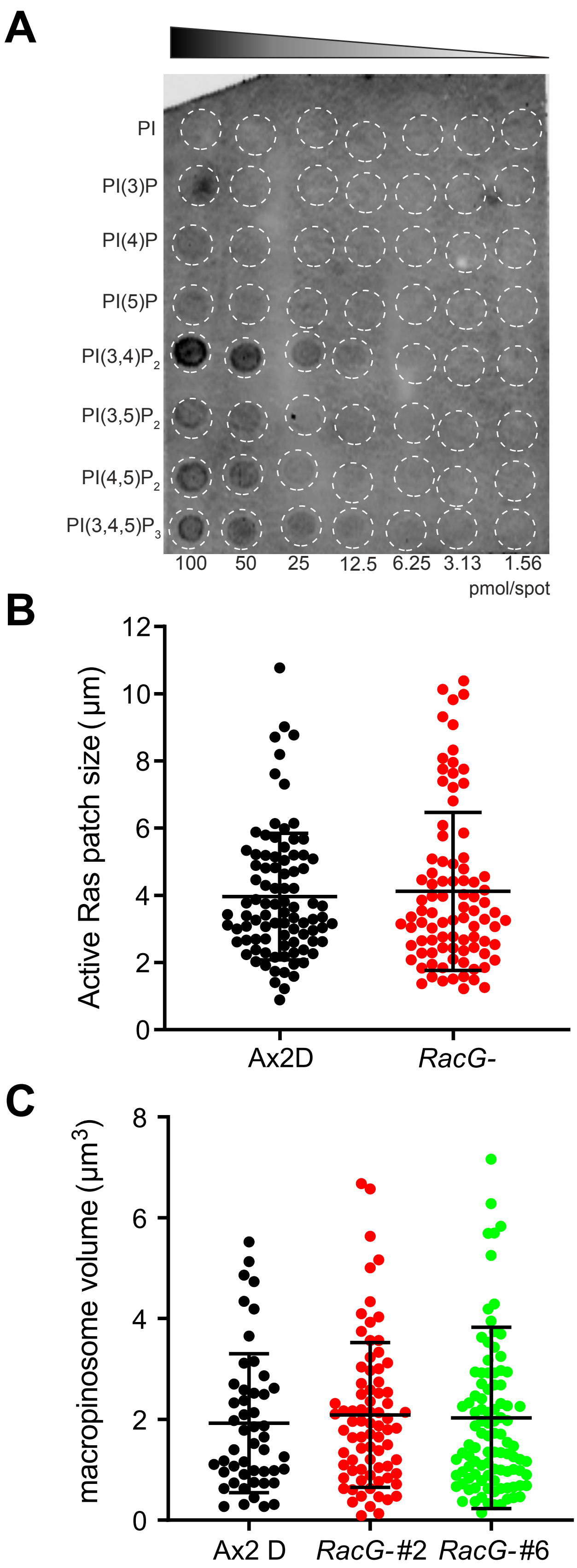

Figure S5: BAR domain lipid binding specificity, and macropinosome formation in RacG mutants, related to Figure 5. (A) PIP array analysis of BAR-GFP binding showing a moderate preference for PI(3,4)P_2 in this assay. (B) Active Ras signaling during macropinosome formation in RacG- cells and their parental cell line Ax2D. Patch size was quantified from single confocal planes of cells expressing RBD-GFP. (C) Volume of macropinosomes formed by RacG- and control cells, measured by imaging cells taking up FITC dextran. Two independent RacG- clones were analysed over 3 independent experiments.
