# Supplementary material for: Coordinated Ras and Rac Activity Shapes Macropinocytic Cups and Enables Phagocytosis of Geometrically Diverse Bacteria
Source: Curr Biol. 2020 Aug 3;30(15):2912–2926.e5. doi: 10.1016/j.cub.2020.05.049 (PMC7416115; doi:10.1016/j.cub.2020.05.049)
Supplement: Document S2. Article plus Supplemental Information [file mmc11.pdf]

Current Biology

Article

Coordinated Ras and Rac Activity Shapes Macropinocytic Cups and Enables Phagocytosis of Geometrically Diverse Bacteria

Graphical Abstract

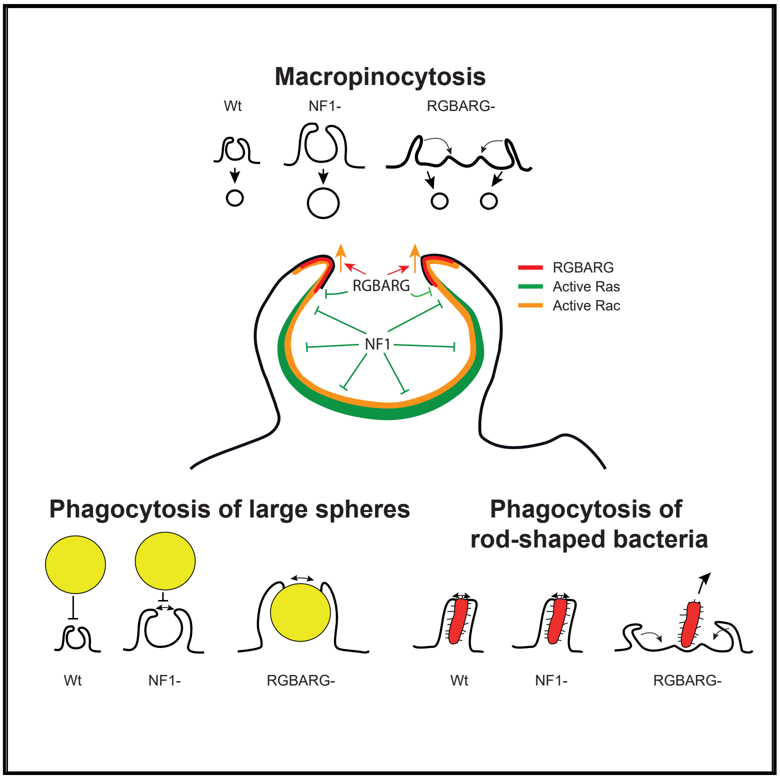

Highlights

- We identify a new regulator that shapes macropinocytic and phagocytic cups
- Shaping protrusions into cups requires differential regulation of Ras and Rac
- Cups are organized by integrating interactions with phospholipids and multiple GTPases
- Defective cup formation causes a target shape-specific defect in phagocytosis

Authors

Catherine M. Buckley, Henderikus Pots, Aurelie Gueho, ..., Andrew J. Parnell, Arjan Kortholt, Jason S. King

Correspondence

jason.king@sheffield.ac.uk

In Brief

Forming cup-shaped protrusions allows cells to engulf extracellular fluid and particles by macropinocytosis and phagocytosis, respectively. Buckley et al. identify a new regulator that differentially regulates small GTPases to generate the cup shape. They propose a model whereby this coordinates the shape and allows cells to engulf different shapes.

Buckley et al., 2020, Current Biology 30, 2912–2926
August 3, 2020 © 2020 The Author(s). Published by Elsevier Inc.
https://doi.org/10.1016/j.cub.2020.05.049

CellPress

CellPress
OPEN ACCESS

Current Biology

Article

Coordinated Ras and Rac Activity Shapes Macropinocytic Cups and Enables Phagocytosis of Geometrically Diverse Bacteria

Catherine M. Buckley,[1,7] Henderikus Pots,[2] Aurelie Gueho,[3,8] James H. Vines,[1] Christopher J. Munn,[1] Ben A. Phillips,[1] Bernd Gilsbach,[4] David Traynor,[5,9] Anton Nikolaev,[1] Thierry Soldati,[3] Andrew J. Parnell,[6] Arjan Kortholt,[2] and Jason S. King[1,10,*]

[1]Department of Biomedical Sciences, University of Sheffield, Sheffield S10 2TT, UK
[2]Department of Cell Biochemistry, University of Groningen, Groningen 9747 AG, Netherlands
[3]Department of Biochemistry, Faculty of Sciences, Sciences II, University of Geneva, CH-1211-Geneva-4, Switzerland
[4]German Centre for Neurodegenerative Diseases, Tübingen 72076, Germany
[5]MRC Laboratory of Molecular Biology, Cambridge CB2 0QH, UK
[6]Department of Physics and Astronomy, University of Sheffield, Sheffield S3 7RH, UK
[7]Present address: Molecular Immunology Unit, Department of Medicine, University of Cambridge, MRC Laboratory of Molecular Biology, Cambridge, CB2 0QH, UK
[8]Present address: Fish Physiology and Genomics Institute, INRA, 35042, Rennes, France
[9]Present address: Cambridge Institute for Medical Research, University of Cambridge, CB2 0XY, UK
[10]Lead Contact
*Correspondence: jason.king@sheffield.ac.uk
https://doi.org/10.1016/j.cub.2020.05.049

SUMMARY

Engulfment of extracellular material by phagocytosis or macropinocytosis depends on the ability of cells to generate specialized cup-shaped protrusions. To effectively capture and internalize their targets, these cups are organized into a ring or ruffle of actin-driven protrusion encircling a non-protrusive interior domain. These functional domains depend on the combined activities of multiple Ras and Rho family small GTPases, but how their activities are integrated and differentially regulated over space and time is unknown. Here, we show that the amoeba *Dictyostelium discoideum* coordinates Ras and Rac activity using the multidomain protein RGBARG (RCC1, RhoGEF, BAR, and RasGAP-containing protein). We find RGBARG uses a tripartite mechanism of Ras, Rac, and phospholipid interactions to localize at the protruding edge and interface with the interior of both macropinocytic and phagocytic cups. There, we propose RGBARG shapes the protrusion by expanding Rac activation at the rim while suppressing expansion of the active Ras interior domain. Consequently, cells lacking RGBARG form enlarged, flat interior domains unable to generate large macropinosomes. During phagocytosis, we find that disruption of RGBARG causes a geometry-specific defect in engulfing rod-shaped bacteria and ellipsoidal beads. This demonstrates the importance of coordinating small GTPase activities during engulfment of more complex shapes and thus the full physiological range of microbes, and how this is achieved in a model professional phagocyte.

INTRODUCTION

The capture and engulfment of extracellular material serves a number of important cellular functions. While the clearance of pathogenic microbes or apoptotic cells by phagocytic immune cells is best understood, the engulfment of fluid by the related process of macropinocytosis also plays important functions by allowing cells to capture antigens or other factors from their environment such as nutrients to support growth [1–6].

To capture extracellular fluid or particulate material, cells must encircle and isolate their target within a vesicle. This can be achieved by several mechanisms, but the mechanism that is best understood and evolutionarily widespread involves the extension of a circular cup- or ruffle-shaped protrusion from the cell surface to enwrap and internalize the target [7–10]. Many components of cup formation have been identified, but how they are coordinated in space and time is poorly understood. Here, we describe a novel mechanism used by the amoebae *Dictyostelium discoideum* to integrate different signaling elements and form complex cup-shaped protrusions that efficiently mediate engulfment.

Macropinocytic and phagocytic protrusions are formed by localized actin polymerization at the plasma membrane, using much of the same machinery that generates pseudopods and lamellipodia during cell migration [10, 11]. While migratory protrusions only need the cell to define a simple patch of actin

 © 2020 The Author(s). Published by Elsevier Inc.
This is an open access article under the CC BY license (http://creativecommons.org/licenses/by/4.0/).

CellPress
OPEN ACCESS

polymerization, forming a cup requires a higher level of organization, with the protrusive activity restricted to a ring encircling a static interior domain. During phagocytosis, this is aided by the presence of a particle to act as a physical scaffold and locally activate receptors. These interactions are proposed to guide engulfment by a zippering mechanism [12, 13]. However, macropinocytic cups self-organize with an almost identical structure in the absence of any external spatial cues [8]. Cup formation can therefore occur spontaneously by the intrinsic dynamics of the underlying signaling.

Recent studies in *Dictyostelium* proposed a model whereby the cup interior is defined by spontaneous localized activation of the small GTPase Ras and consequent accumulation of the phospholipid PIP_3 [8]. This patch appears to restrict actin polymerization to its periphery to create a protrusive ring. How this is achieved is unknown, but in at least *Dictyostelium*, it may depend on the activity of the PIP_3-activated protein kinase B/Akt [14]. Both active Ras and PIP_3 also accumulate at cups in mammalian cells [15–17], and Ras activation is sufficient to drive ruffling and macropinocytosis in cancer cells [5, 18]. PI3K inhibition also completely blocks macropinocytosis [19–22] and phagocytosis of large particles by macrophages [22–24]. Ras and PIP_3 therefore play a general role in macropinosome and phagosome organization across evolution.

Other small GTPases are also involved in cup formation. Active Rac1 overlaps with Ras activity in the cup interior in both macrophages and *Dictyostelium* [8, 25]. Rac1 is a direct activator of the SCAR/WAVE complex, which drives activation of actin polymerization via the ARP2/3 complex [26, 27]. Consistent with this, Rac1 is required for macropinosome formation in dendritic cells [28], and optogenetic Rac1 activation is sufficient to drive ruffling and macropinocytosis in macrophages [29]. Expression of constitutively active Rac1 also leads to excessive actin at macropinocytic cups in *Dictyostelium* [30]. Therefore, while Ras appears to define the cup interior, Rac1 is important for regulating actin protrusions, as it is does during cell migration.

The presence of active Rac1 throughout the cup interior is at odds with the tightly restricted SCAR/WAVE activity and protrusion at the extending rim [8]. Therefore, further layer of regulation must exist. This is likely provided by the small GTPase CDC42 that is also required for Fc-γ-receptor-mediated phagocytosis and collaborates with Rac1 during engulfment of large particles [23, 31–33]. In contrast to Rac1, active CDC42 is restricted to the protrusive cup rim in macrophages indicating differential regulation and functionality [25]. In *Dictyostelium* however, no clear CDC42 ortholog has been identified.

Cup formation requires integrated spatio-temporal control over multiple GTPases. This must be able to self-organize in the absence of external cues during macropinocytosis and robust enough to phagocytose physiological targets of varying size and shape. Small GTPase activity is controlled by a large family of proteins such as GTPase exchange factors (GEFs), which promote the GTP-bound active form, and GTPase activating proteins (GAPs), which stimulate hydrolysis and transition to a GDP-bound inactive state. In this study, we characterize a previously unstudied dual GEF- and GAP-domain-containing protein in *Dictyostelium* that integrates Ras, Rac, and lipid signaling. This provides a mechanism to coordinate the cup interior with the protrusive rim, allowing efficient macropinosome formation and the engulfment of diverse bacteria of differing geometry.

RESULTS

Identification of a Novel BAR-Domain-Containing Protein Recruited to Cups

Our initial hypothesis was that cells may use the different membrane curvature at the protrusive rim compared to the cup base to recognize and differentially regulate cup shape. Membrane curvature can recruit proteins containing BAR (Bin-Amphiphysin-Rvs) domains [34]. To identify candidates involved in macropinocytosis, we searched the *Dictyostelium* genome for BAR domain-containing proteins. Excluding proteins of known localization or function, we systematically cloned each candidate and expressed them as both N- and C-terminal GFP-fusions in axenic Ax2 cells. We thus cloned nine previously uncharacterized BAR-containing proteins and observed their localization in live cells. Of these, six were expressed at detectable levels (Figure 1A).

DDB_G0284997, DDB_G0305372, and DDB_G0285851 were associated with plasma membrane puncta, consistent with the well-characterized role of BAR domains in clathrin-mediated endocytosis [35]. DDB_G0276447 localized to vesicles too small to be macropinosomes, and GFP-DDB_G0272368 was exclusively in the nucleus. Only one of the proteins tested (DDB_G0269934) localized to what appeared to be the protrusive regions of macropinocytic cups.

DDB_G0269934 is a 225 kDa multidomain protein and also contains regulator of chromatin condensation (RCC1), RhoGEF, and RasGAP domains (Figure 1B). DDB_G0269934 has not previously been studied, and due to its domain organization, we will subsequently refer to it as RGBARG (RCC1, GEF, BAR and GAP domain-containing protein, encoded by the *rgbA* gene). Combining BAR, GEF, and GAP activities in a single protein potentially provides an elegant mechanism to coordinate Ras and Rac activity to organize engulfment. We therefore investigated RGBARG in detail.

Examining RGBARG-GFP dynamics by time-lapse microscopy confirmed strong enrichment at the protrusive rim and weaker enrichment in the interior of both macropinocytic and phagocytic cups, delocalizing rapidly after engulfment (Figures 1C and 1D; Videos S1 and S2). Co-expression with the PIP_3 reporter PH_{CRAC} -RFP that demarks the cup interior [36] confirmed RGBARG-GFP localized to the periphery of this signaling domain (Figures 1E–1H; Video S3). This differs from the RasGAP Neurofibromin (NF1), which was previously reported to control cup formation, and localizes throughout the cup [37]. RGBARG may therefore play a specific role in organizing engulfment.

RGBARG and NF1 Play Distinct Roles in Macropinosome Formation

To test its functional role, we disrupted the *rgbA* locus, deleting a 3.6 Kb region of the middle of the gene (Figure S1). Independent clones were isolated (JSK02 and 03) with comparable phenotypes. JSK02 was used unless otherwise stated with effects of loss of RGBARG validated by rescue experiments.

To check for macropinocytic defects, cells were incubated with FITC-dextran, a pH sensitive dye that is quenched at low

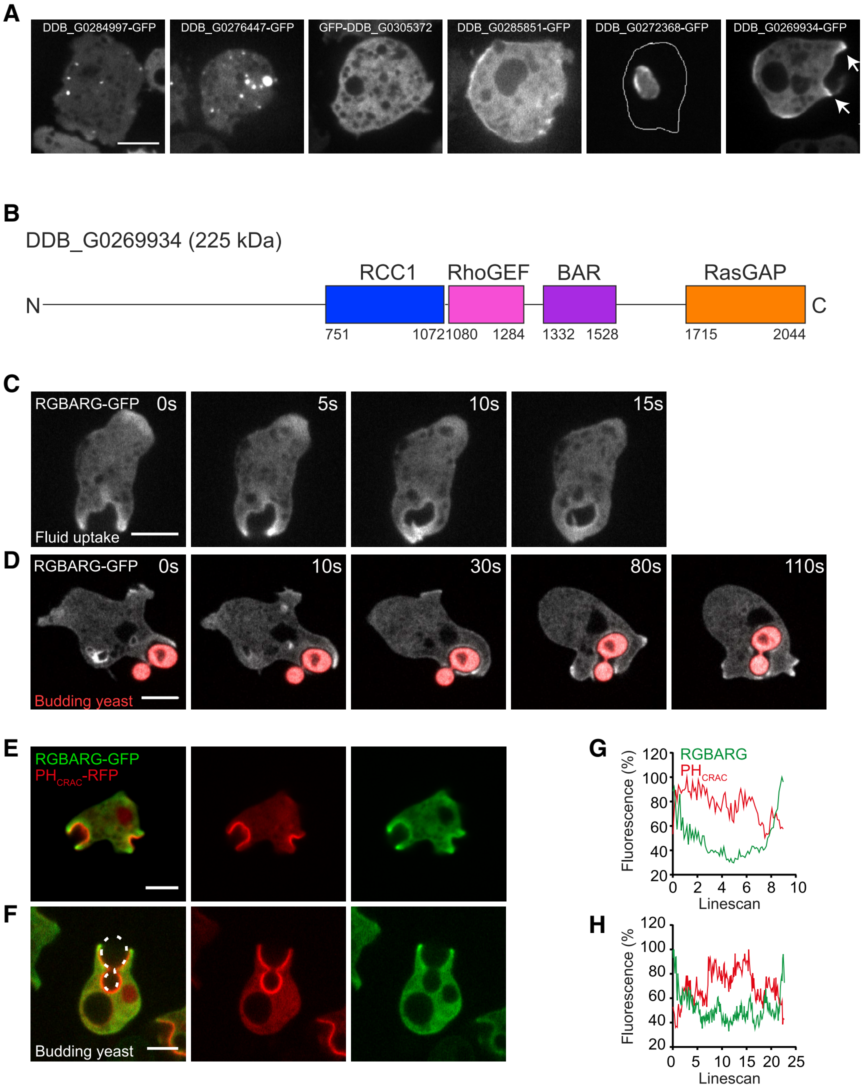

Figure 1. Identification of BAR Domain Proteins Associated with Macropinocytosis

(A) Localization of BAR-domain proteins expressed as GFP-fusions in Ax2 cells, maximum intensity projections of confocal stacks.

(B) The domain organization of DDB_G0269934/RGBARG.

(C and D) Time series of RGBARG-GFP during macropinocytosis (C) and phagocytosis (D) of TRITC-labeled yeast (Video S1 and Video S2).

(E and F) RGBARG-GFP localization relative to PH_{CRAC}-RFP (PIP_3) during (E) macropinocytosis and (F) phagocytosis. A 3D-timelapse is shown in Video S3.

(G and H) The intensity profiles of each protein from linescans along the cup interior from tip to tip. (G) corresponds to the macropinocytic cup in (E) and (H) to the phagocytosis in (F). Scale bars, 5 μm

pH. As *Dictyostelium* macropinosomes acidify in under two min, only nascent macropinosomes are visible (Figure 2A). In this assay, RGBARG− cells formed slightly more, but significantly smaller macropinosomes, measuring 0.5 ± 0.1 μm^3 compared to 1.5 ± 0.2 μm^3 in Ax2 controls (Figures 2B and 2C). Consistent with this, bulk fluid uptake was reduced by ~50% in RGBARG− cells, although this did not significantly inhibit axenic growth (Figures 2D and 2E).

As engulfment and migration use much of the same machinery, we also tested chemotaxis toward folate. While chemotactic accuracy was unaltered, RGBARG− cells only moved at half the speed of Ax2 controls (6.3 μm/min versus 13.9 μm/min; Figures S1C–S1E; Video S4), migrating with an exceptionally broad and persistent leading edge (Video S5). In contrast to the clear enrichment at cup protrusions, however, very little RGBARG-GFP was recruited to the leading edge of chemotaxing cells, with only small, transient puncta visible (Figure S1F). We therefore focused on the role of RGBARG in cup formation.

The mutants above were generated in the Ax2 laboratory strain, which harbors a mutation in the *axeB* gene encoding NF1 that facilitates axenic growth by increasing fluid uptake [37]. To test how RGBARG affects macropinocytosis in cells with intact NF1, we made additional mutants in the wild-type DdB strain, named JSK18 and 19. We refer to both Ax2 and DdB background mutants as RGBARG− cells, which are identified by the appropriate parental control.

Using DdB-derived NF1 mutants [37] we compared the effect of disrupting RGBARG or NF1 alone. NF1 loss increased macropinosome volume over 2-fold with no change in number, but DdB-derived RGBARG mutants again formed larger numbers of significantly smaller vesicles (Figures 2F and 2G). This had no significant effect on total fluid uptake, and consequently was unable to enhance axenic growth (Figures 2H and 2I). RGBARG is therefore functionally important during macropinosome formation and plays a distinct role to NF1.

RGBARG Coordinates Signaling and Regulates Cup Shape

As both RGBARG and NF1 are RasGAPs, we examined Ras signaling using the Ras binding domain of Raf1 fused to GFP (GFP-RBD). Disruption of NF1 in DdB cells increased the average active Ras domain from 5.6 μm to 6.5 μm (Figures 3A and 3B). This affect is more modest than previously described because we axenically adapted DdB-derived strains in medium enriched with 20% serum prior to all experiments, rather than unsupplemented medium [37]. Under these conditions, DdB cells better overcome the suppression of macropinocytosis by bacteria or starvation [38] and take up more fluid in significantly larger cups. Although neither mutant altered the number of active Ras patches, disruption of RGBARG had a much larger effect on patch size, averaging 13.4 μm, encompassing 55% of the entire cell perimeter, compared with 28% and 29% for DdB and NF1− cells, respectively (Figures 3A–3D). Similar results were obtained

CellPress
OPEN ACCESS

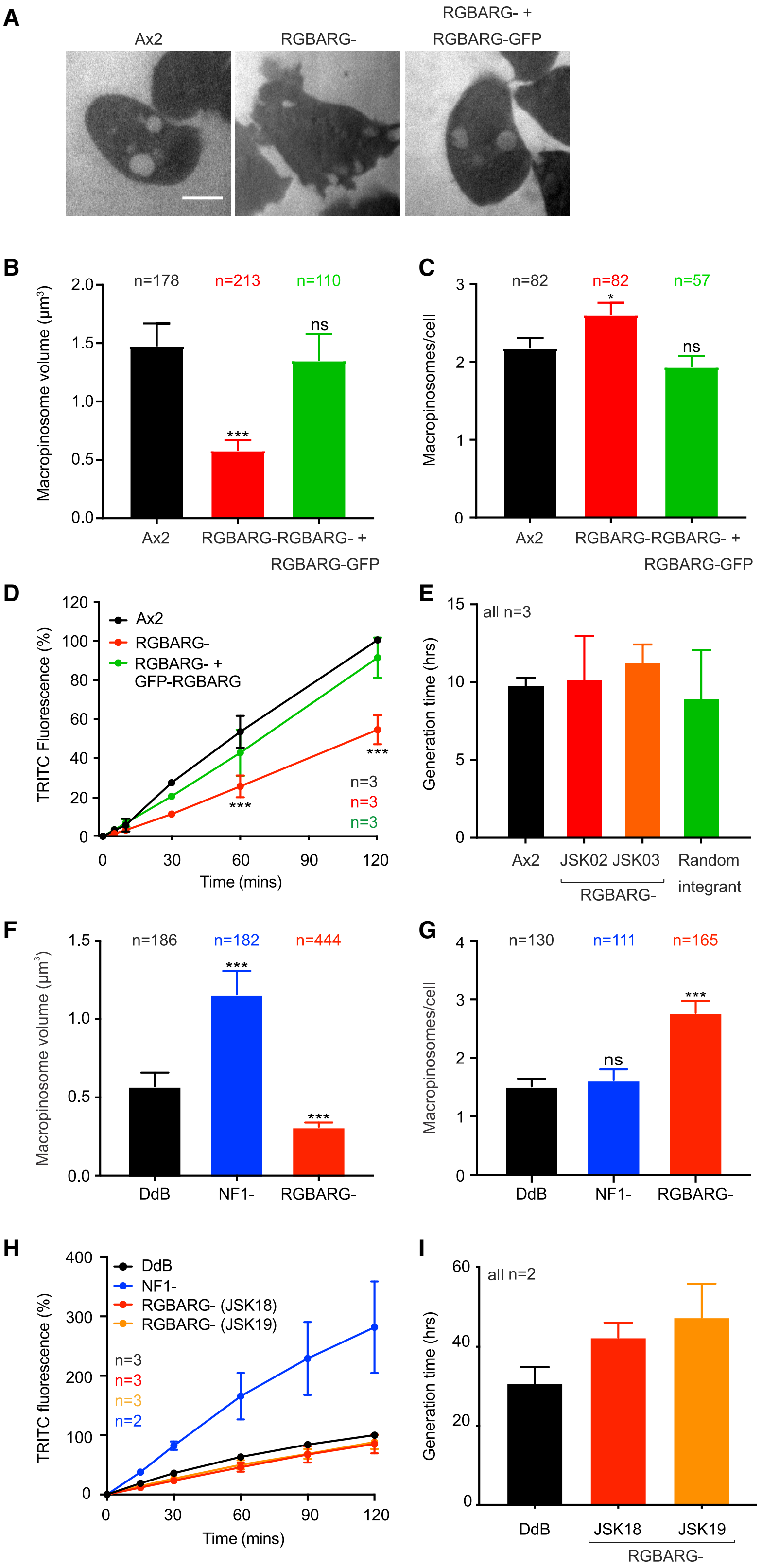

Figure 2. RGBARG– Cells Produce More, but Smaller, Macropinosomes

(A) Confocal images of cells incubated in FITC-dextran for 10 min. pH-sensitive FITC is only visible <2 min after engulfment. Scale bar represents 5 µm.

(B and C) Average macropinosome volume (B) and frequency (C) per cell. n = total number of macropinosomes or cells measured over 3 experiments.

(D) Total fluid uptake, measured by TRITC dextran uptake, measured by flow cytometry.

(E) Growth of RGBARG– cells in HL5 medium compared to Ax2 parents and a random integrant control.

(F and G) Macropinosome (F) volume and (G) number in the non-axenic DdB strain and isogenic NF1 and RGBARG mutants, measured as in (B) and (C).

(H) Total TRITC-dextran uptake, measured by flow cytometry.

(I) Growth of DdB -derived RGBARG– mutants (JSK18 and 19) in HL5 medium + 20% FCS.

All experiments were performed on adherent cells. Graphs show means ± SEM; *p < 0.05, ***p < 0.001, determined by Mann-Whitney t test. Related Figure S2 and Videos S4 and S5 show mutant generation and analysis of chemotaxis.

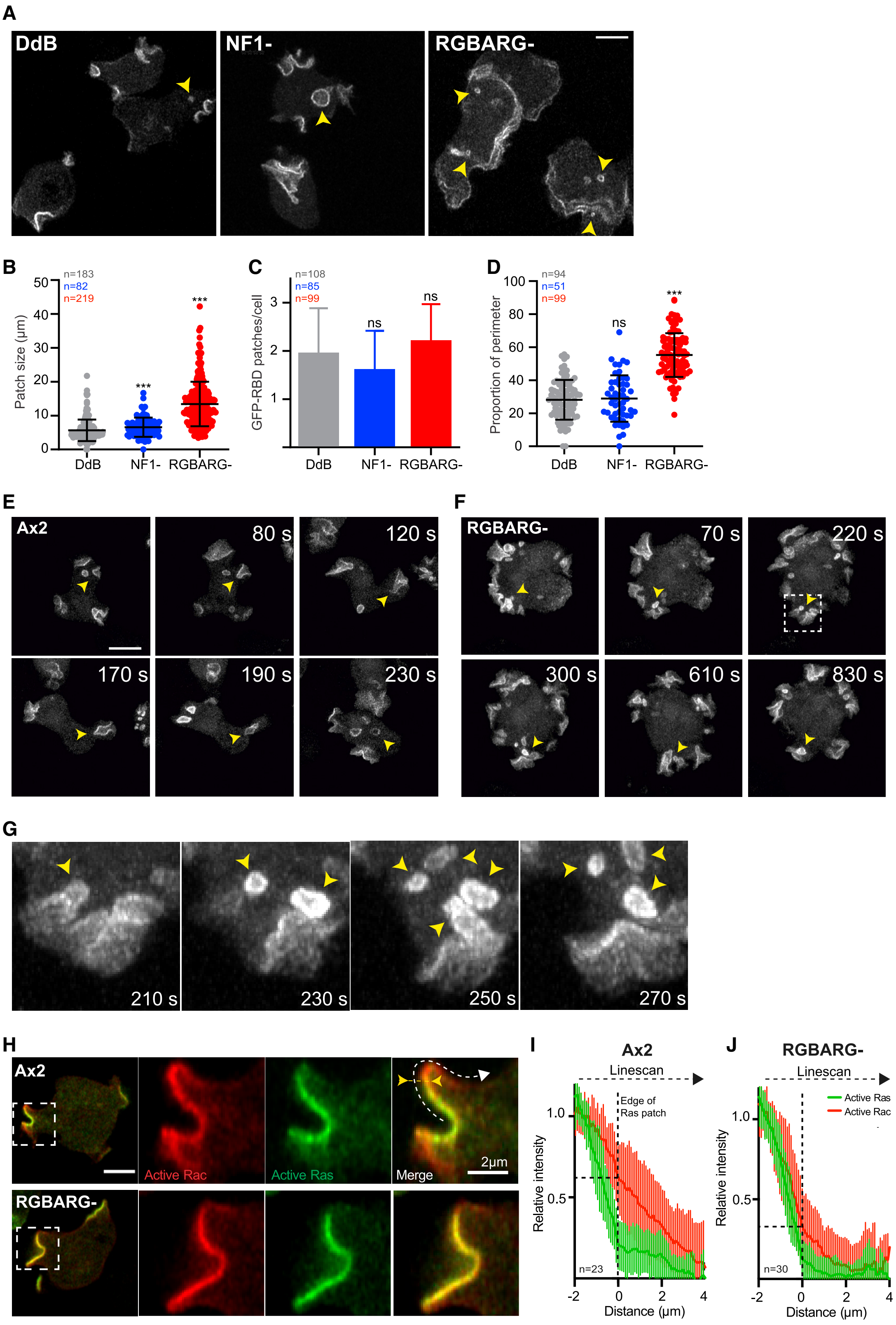

Figure 3. RGBARG Regulates Cup Dynamics

(A) Maximum intensity projections of DdB-derived mutants expressing GFP-RBD (from Video S7). Arrows indicate completed vesicles at internalisation.
(B and C) Active Ras patch size (B) and frequency (C) in single planes through the cell center.

(legend continued on next page)

CellPress
OPEN ACCESS

with both active Ras and PIP_3 probes in Ax2-derived RGBARG mutants, although in these cells the number of patches was increased 2-fold (Figures S2A–S2E).

To understand how the enlarged Ras and PIP_3 patches in RGBARG− cells give rise to smaller macropinosomes, we studied three-dimensional (3D) signaling dynamics over time. As described in Ax2 cells, macropinocytic cups form by expanding around a spontaneous patch of PIP_3 [8]. These subsequently close, usually forming 1–2 large macropinosomes accompanied by termination of PIP_3 signaling on both the new vesicle and the cell surface (Figure 3E; Video S6). This process is relatively consistent, with each PIP_3 patch lasting an average of 150 s (Figure S2F).

In Ax2 RGBARG− cells, PH_{CRAC}-GFP still disappeared from internalized vesicles, but the plasma membrane domains were much flatter and more stable; while PIP_3 patches frequently split, they rarely dissipated completely and often lasted longer than the 30 min videos (Figure 3F; Video S6). It was therefore not possible to meaningfully measure the lifetime of PIP_3 signaling in RGBARG− cells. An identical phenotype was observed in DdB-derived RGBARG mutants expressing GFP-RBD. In contrast, the cups formed by NF1− cells were similar in shape to controls but larger, forming over a longer time with surface signaling terminating at closure (Video S7). RGBARG therefore appears to primarily regulate cup shape, whereas NF1 regulates its size.

Although extinction of signaling did not accompany closure in RGBARG− cells, numerous small vesicles continuously budded from the ruffle base when folds of membrane collapsed in on themselves (Figure 3G; Videos S6 and S7). This explains why these cells form more frequent but smaller macropinosomes, corroborating our FITC-dextran data (Figures 2, S2G, and S2H). This formation mechanism implies that the entire PIP_3 patch is potentially fusogenic and can internalize vesicles by simply folding onto itself rather than requiring a specific mechanism for closure and fission at the rim.

The RasGAP and RhoGEF domains of RGBARG could potentially coordinate both Ras and Rac signaling. We therefore co-expressed probes for the active forms of both small GTPases to study their activities relative to each other. In Ax2 cells, the active Ras probe was restricted inside the cup rim, whereas active Rac recruitment also encompassed the protrusive edge, extending up to 2 μm further (Figures 3H–3J). This differential was lost in RGBARG− cells, with both Ras and Rac probes restricted to within the rim (Figure 3J). Although potential differences in probe affinity mean we cannot be sure of the precise extent of each signaling domain, this shows RGBARG differentially regulates Ras and Rac and spatially coordinates their activities.

To test whether loss of RGBARG generally affected cup organization, we also expressed GFP-fusions of the class I myosin myoIB, which exhibits a similar rim enrichment to RGBARG; the SCAR/WAVE complex, which drives actin polymerization at the protrusive cup rim; and PTEN (phosphatase and tensin homolog), which degrades PIP_3 and is excluded from cups. All three proteins localized normally in RGBARG mutants, indicating RGBARG controls cup dynamics, rather than being required to recruit specific effectors (Figures S2I–S2K).

GEF, GAP, and BAR Domain Interactions Each Contribute to RGBARG Positioning

RGBARG localization will be critical to position its RhoGEF activity where protrusion is promoted, and the RasGAP activity where it can restrain expansion of the interior. To dissect the mechanisms of RGBARG recruitment, we tested the effect of deleting each protein domain in turn (Figure S3). To quantify RGBARG enrichment across the cup, line scans from cup tip to tip were averaged across multiple cells. GFP-fused to the cyclic AMP receptor (cAR1-GFP) localizes uniformly to the plasma membrane and was used as a control (Figures 4A–4D). This method confirmed RGBARG-GFP was enriched 3-fold at the protruding edges and 2-fold at the cup base, allowing us to quantify how each domain contributes to recruitment at the cup.

Removal of the RCC1 domain had no effect on localization and fully rescued the formation of large macropinosomes (Figures 4E–4H). In contrast, deletion of either the RhoGEF or BAR domains caused RGBARG to become uniformly cytosolic and did not rescue the defect (Figures 4E–4H). RGBARGΔGAP-GFP however still localized to the plasma membrane but was much more broadly distributed throughout the cup. Consequently, it was significantly less enriched at the rim and unable to rescue the mutant defect in macropinosome formation (Figures 4E–4H). Co-expression with PH_{CRAC}-RFP confirmed RGBARGΔGAP-GFP was no longer excluded from PIP_3/active Ras domains (Figure S4). RasGAP interactions therefore restrict RGBARG to the periphery of the cup interior.

To confirm the role of the RasGAP interactions in restricting RGBARG localization, we also made a point mutation in the conserved arginine that stabilizes the Ras-GTP to Ras-GDP transition [39]. This mutation (R1792K) is predicted to disrupt GAP activity but still allow Ras binding. $RGBARG_{R1792K}$-GFP was reduced in the cup interior similar to the wild-type protein and was slightly more enriched at the cup tip (Figures 4F, 4G, and S4B). However, despite recruitment to the protruding rim, $RGBARG_{R1792K}$-GFP did not rescue cup organization of RGBARG− cells, which still produced enlarged PIP_3 patches and small macropinosomes (Figures 4H and S4D). The RasGAP domain of RGBARG therefore also provides spatial information to position RGBARG to the periphery of the active Ras/PIP_3 patch.

(D) Shows the same data, as the proportion of the cell perimeter.

(E and F) Time series of 3D projections through (E) Ax2 and (F) RGBARG− cells expressing PH_{CRAC}-GFP (Video S6). Arrows indicate newly internalized vesicles.

(G) Is an enlargement of the boxed region in (F). See Figure S2 for analysis of Ras/PIP_3 signaling in Ax2 mutants.

(H) Relative localization of Ras and Rac activity, using GFP-RBD and RFP-PakB-CRIB respectively.

(I and J) Quantification of Rac activity relative to the Ras patch edge in (I) Ax2 and (J) RGBARG− cells, averaged over multiple cups. Intensity profiles were measured as per the dotted arrow in (H) and aligned to the active Ras patch edge (yellow arrowheads and line, 0 μm on the x axis).

n = total number of cells or patches over 3 independent experiments. Error bars denote mean ± standard deviation; ** $p < 0.01$, *** $p < 0.001$, Mann-Whitney t test. Scale bars, 5 μm unless otherwise indicated.

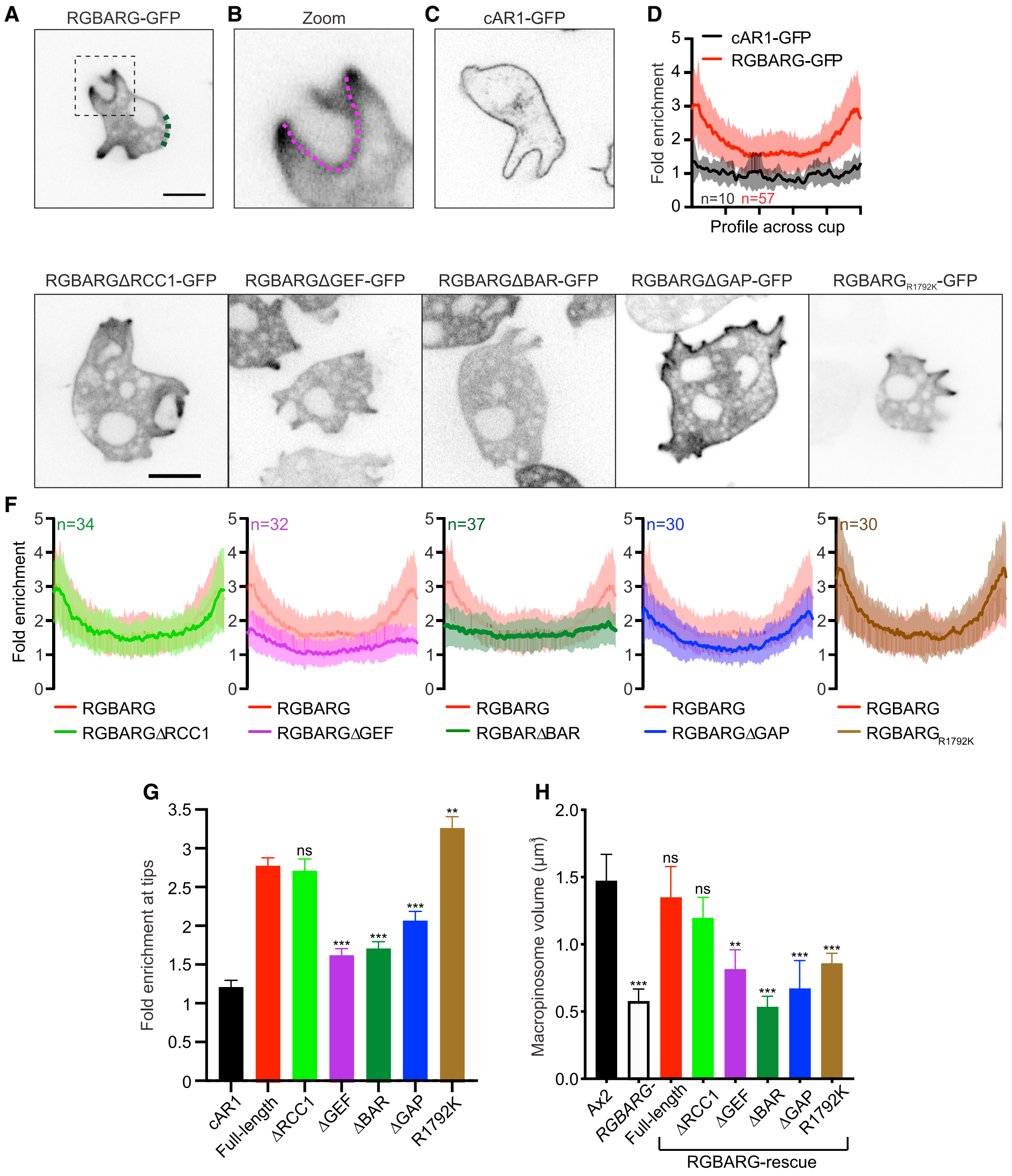

Figure 4. Multiple Interactions Regulate RGBARG Recruitment

Full-length RGBARG or mutants lacking individual domains were expressed as GFP-fusions in (Ax2) RGBARG– cells. Truncations are shown in Figure S3.

(A) Shows full-length RGBARG-GFP and enrichment relative to a non-protrusive region (green dotted line).

(B) Enlargement of the boxed region, showing an example linescan measured across the cup interior.

(C) The uniform localization of cAR1-GFP control.

(D) Averaged, normalized linescans from multiple cells, demonstrating RGBARG-GFP enrichment at the cup rim.

(E) Representative images of RGBARG truncation mutants, as well as the RasGAP-inactivating R1792K point mutant (further analyzed in Figure S4).

(F) Averaged intensity of each construct across the cup, compared to the full-length protein from (D), in red. Values plotted are the mean ± standard deviation.

(G) Rim-enrichment of each construct, measured by averaging the first 10% of each linescan.

(H) The ability of each construct to rescue large macropinosome formation in RGBARG– cells determined by the size of FITC dextran-containing macropinosomes.

>100 macropinosomes over three experiments were measured. Bars denote mean volume ± SEM, $^{**}p < 0.01$, $^{***}p < 0.005$ Mann-Whitney t test. Scale bars represent 5 μm

To identify the relevant binding partners and contribution of each domain, we also expressed them individually, fused to GFP. Although RhoGEF-GFP expressed too poorly to observe its localization, both the RCC1 and GAP domains expressed well and were completely cytosolic (Figures 5A and 5C). In contrast, the BAR domain alone was sufficient for strong recruitment throughout the plasma membrane (Figure 5B). This was blocked by including either of the adjacent RhoGEF or RasGAP domains (Figures 5D and 5E), indicating additional intramolecular interactions. In contradiction to our initial hypothesis, however, BAR-GFP was not enriched at areas of curvature or protrusion. The BAR domain therefore appears to drive general recruitment to the plasma membrane rather than recognize curvature at cups.

As the BAR domain does not concentrate at specific membrane shapes, we investigated its lipid binding specificity by lipid-protein overlay. PIP strips indicated BAR-GFP bound to all PIPs with two or more phosphates (Figure 5F), with PIP arrays indicating a slight selectivity for PI(3,4)P_2 (Figure S5A). This broad binding to all highly phosphorylated phosphoinositides indicates that this BAR domain is likely to generally recognize high negative charge rather than specific phosphate configurations.

To identify the targets of the RhoGEF domain, we performed co-immunoprecipitations with a library of recombinant GST-tagged small GTPases. The *Dictyostelium* genome contains an expanded set of Rac small GTPases, but no Rho or CDC42 subfamily members [40]. Of these, only RacH and RacG bound the RhoGEF domain of RGBARG with no detectable binding to other Racs, including Rac1, which has previously been implicated in cup formation [30].

While RacH is involved primarily in endocytic trafficking and localizes exclusively to intracellular compartments [41], RacG localizes to the plasma membrane and is enriched at the protruding rim of phagocytic cups [42]. Overexpression of wild-type or constitutively active RacG also promotes phagocytosis [42], indicating a potential interaction with RGBARG.

Consistent with previous reports [42], RacG mutants had no significant macropinocytic defect, forming normal sized active Ras patches and macropinosomes (Figures S5B and S5C). RGBARG-GFP recruitment to cups however was more uniform and only enriched 1.8 ± 0.6-fold at the rim of RacG- cells compared with 2.6 ± 0.7-fold in isogenic controls (Figures 5H–5L). This indicates that RacG and RGBARG functionally interact *in vivo* and partly contribute to RGBARG localization. However, redundancy with other Racs or additional interactions are sufficient for partial RGBARG recruitment and apparently normal engulfment in the absence of RacG.

Combined, our data indicate that RGBARG uses a coincidence detection mechanism to direct cup formation: BAR domain binding to negatively charged phospholipids directs the protein to the plasma membrane while additional interactions with RacG and active Ras constrain RGBARG to the cup rim. This tripartite regulation ensures that RGBARG is accurately positioned to exert its RhoGEF and RasGAP activities at the interface between cup interior and protrusion to organize engulfment.

RGBARG Is a Highly Active Dual Specificity Ras/Rap GAP

To investigate the differences between NF1 and RGBARG, we compared the specificity and activities of their RasGAP domains. The *Dictyostelium* genome encodes 14 Ras subfamily members of which RasB, RasG, and RasS are the most important for macropinocytosis [20, 43–45]. Overexpression of RasD can also partially compensate for loss of RasG and S [45]. The small GTPase Rap, a close relative of Ras, has also been implicated in macropinosome formation [46]. We therefore measured the GAP activities of both NF1 and RGBARG against each small GTPase.

Consistent with the inability of $\text{RGBARG}_{\text{R1792K}}$-GFP to rescue the knockout, the RGBARG RasGAP domain was active against all GTPases tested (Figures 5M and 5N). The RasGAP domain of NF1 was also active against each Ras but with 75% less activity than RGBARG in each case. RGBARG is therefore a more potent RasGAP *in vitro*, but the lack of specificity for particular Ras isoforms for both RGBARG and NF1 indicates their functional differences are imparted by localization and dynamics.

Loss of RGBARG Improves Phagocytosis of Large Objects

As engulfment of solid particles such as microbes uses much of the same machinery as macropinocytosis and RGBARG also localizes to phagocytic cups, we also investigated how RGBARG contributes to phagocytosis. Disruption of NF1 was previously shown to increase the size of particles that *Dictyostelium* can engulf [37]. As RGBARG also restricts the PIP_3 domains that define the cup interior, we first tested the ability of Ax2 RGBARG− cells to phagocytose different sized beads. Although disruption of RGBARG had no effect on phagocytosis of 1 μm diameter beads, engulfment of 4.5 μm beads was significantly enhanced with an average of 2.2 ± 0.4 beads engulfed per cell after 1 h, compared with 1.0 ± 0.4 in Ax2 (Figures 6A and 6B). Enhanced Ras activation therefore appears generally beneficial for the engulfment of large beads.

Surprisingly, although extrachromosomal expression of RGBARG-GFP fully rescued macropinosome formation (Figures 2A–2D), it reduced the ability of RGBARG− cells to engulf 4.5 μm beads to 63% of control levels (Figure 6B). This effect was even more severe with domain deletion constructs including the ΔBAR, ΔGEF, and ΔGAP constructs, which do not localize properly and have no deleterious effect on macropinosome formation. This indicates a dominant negative effect, most likely due to sequestration of binding partners by overexpressed and/or mislocalized protein. In contrast, expression of $\text{RGBARG}_{\text{R1792K}}$ had no inhibitory effect on RGBARG− cells. This only differs from RGBARG-GFP in its RasGAP activity indicating that mislocalized or overexpressed RasGAP activity is sufficient to inhibit engulfment of large targets.

To better understand how loss of RGBARG affects phagocytosis, we observed engulfment of TRITC-labeled yeast by cells expressing PH_{CRAC}-GFP. Engulfment occurred rapidly in both cell types but failed at a frequency of 22% ± 7% in Ax2 cells with the PIP_3 patch dissipating and the yeast escaping (Figure 6C; Video S8). While the time for successful engulfment was similar upon loss of RGBARG (129 ± 11 s in mutants versus 147 ± 18 s in Ax2), capture was much more robust failing in only 4% ± 5% of attempts (Figures 6D–6F; Video S9). The main influence of RGBARG on the phagocytic efficiency of large targets thus appears to be increased cup stability and enlarged Ras signaling rather than rate of protrusion around the object.

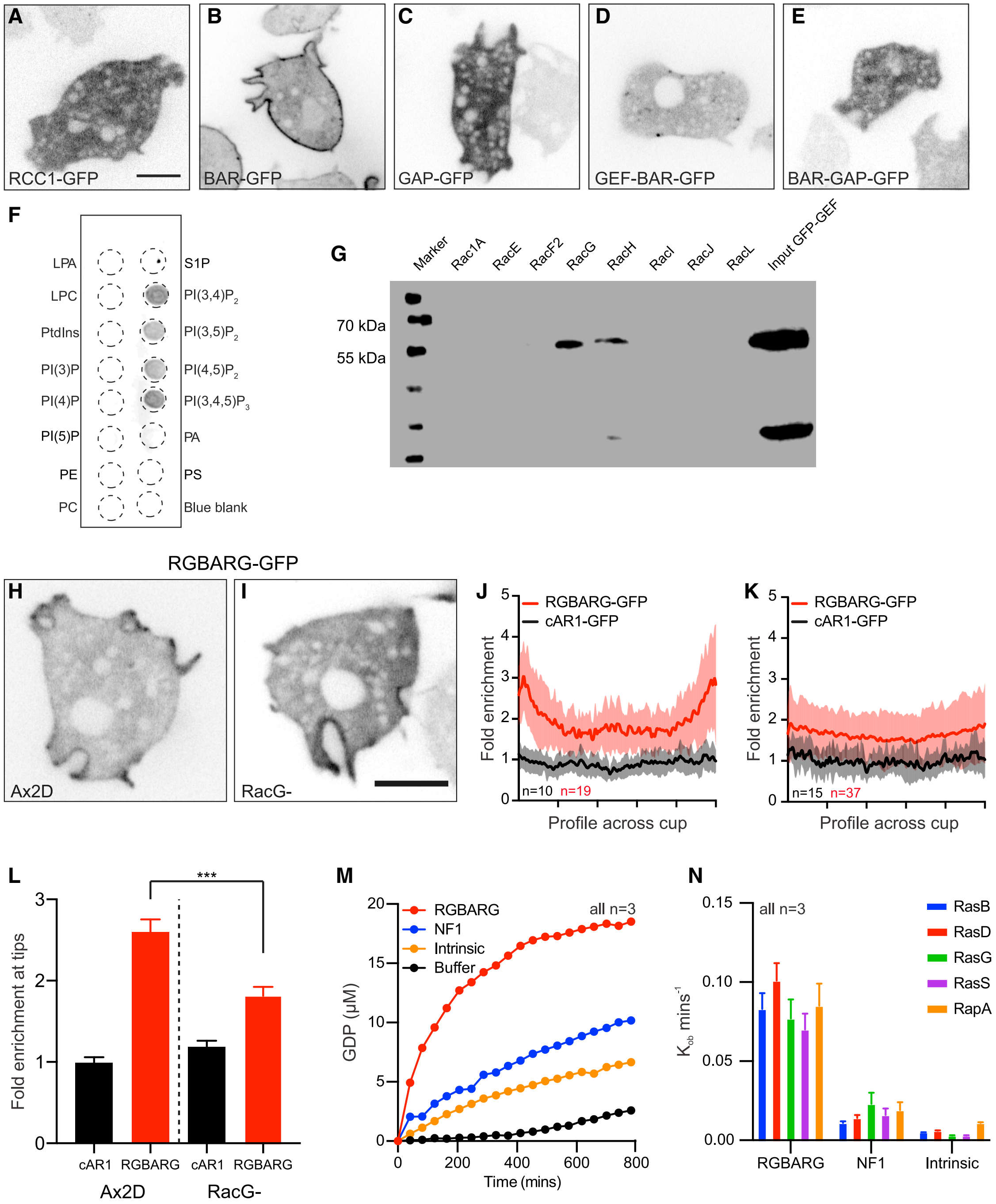

Figure 5. BAR, GEF, and GAP Domain Specificity

(A–E) Single confocal sections of the (A) RCC1, (B) BAR, (C) GAP, (D) GEF and BAR, and (E) BAR and GAP domains of RGBARG domains expressed as GFP fusions in (Ax2) RGBARG– cells (construct details shown in Figure S3).

(F) Lipid overlay assay using whole cell lysate from cells expressing BAR-GFP, PIP array data are shown in Figure S5.

(G) Co-immunoprecipitation of GEF-GFP against a library of purified GST-Rac's bound to beads.

(H and I) Confocal images of full-length RGBARG-GFP in (H) the Ax2D parental cell line and (I) RacG– mutants.

(J and K) Average profile (±standard deviation) of RGBARG-GFP along the cup relative to cAR1-GFP in (J) Ax3D and (K) RacG– cells.

(L) Shows enrichment at cup tips in each cell line. Bars indicate mean ± SEM, ***$p < 0.005$ Mann-Whitney t test. Further analysis of *RacG-* macropinocytosis is shown in Figure S5.

(M) GDP released from GTP-loaded RasG upon addition of the recombinant RasGAP domains from RGBARG and NF1, compared with intrinsic GAP activity or GTP in buffer.

(N) GAP activity of NF1 and RGBARG against a library of Ras superfamily members, performed as in (M) in parallel.

Bars indicate mean ± standard deviation, all scale bars indicate 5 μm.

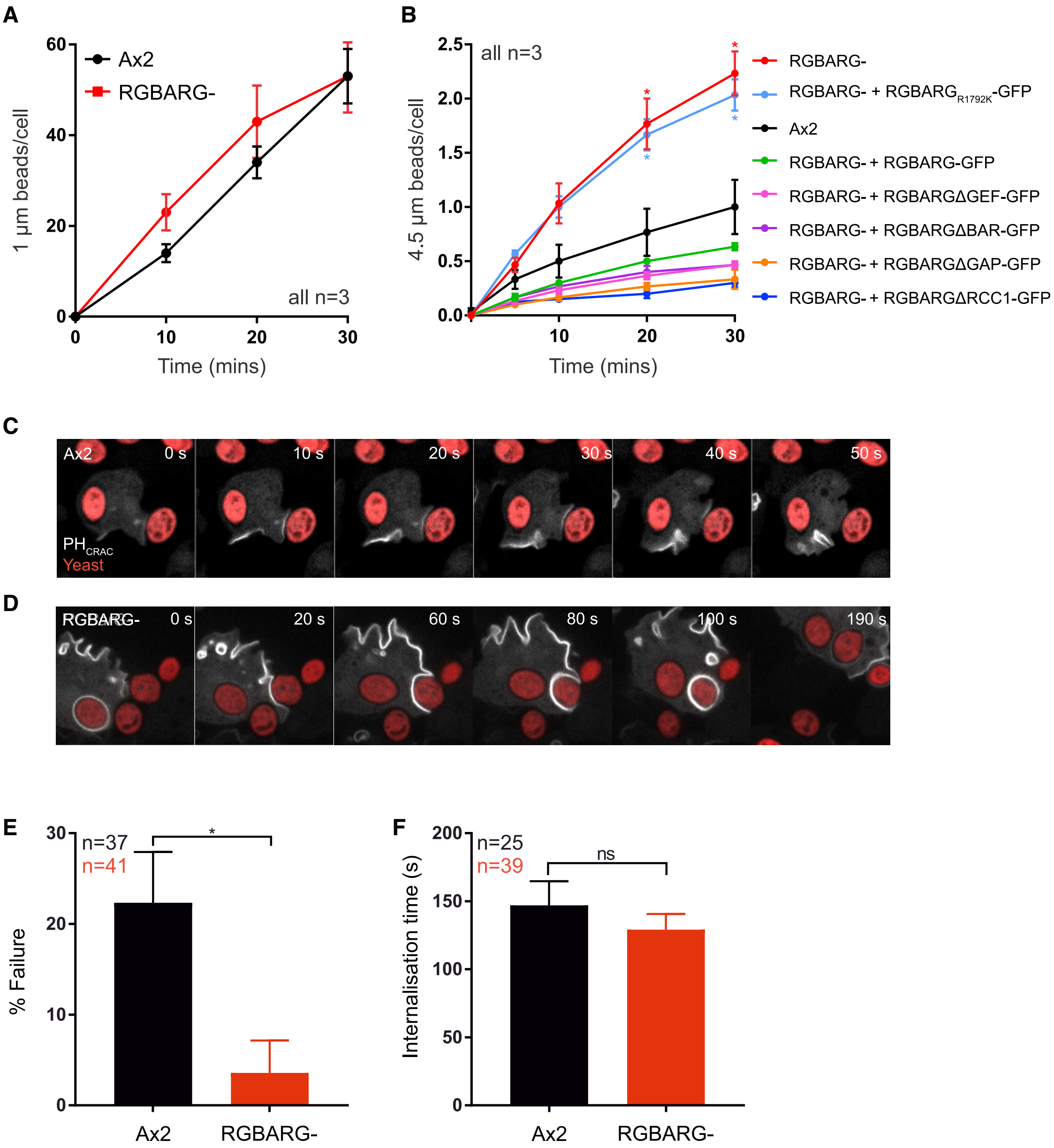

Figure 6. Phagocytic Defects in RGBARG– Cells

(A and B) Phagocytosis of 1.0 or 4.5 µm beads respectively by Ax2, RGBARG–, or RGBARG– cells expressing full-length or mutant RGBARG-GFP.

(C and D) Phagocytosis of TRITC-labeled yeast by cells expressing PH_{CRAC}-GFP observed by spinning disc microscopy. (C) Shows failed engulfment by an Ax2 cell, (D) shows successful engulfment by an RGBARG– cell (Videos S8 and S9).

(E) Relative frequency of phagocytosis failure after cup formation (indicated by PH_{CRAC}-GFP recruitment).

(F) Time from initial contact to completed engulfment in successful phagocytosis.

n = total phagocytic events over three independent experiments. All values are mean ± standard deviation. *$p < 0.01$ unpaired t test.

Spatial Regulation of Ras by RGBARG Is Important for Phagocytosis of Elongated Targets

Phagocytic cells must engulf microbes with differing physical properties such as shape, size, stiffness, and surface chemistry. As RGBARG is important for phagocytic and macropinocytic cup organization, we investigated its role during engulfment of different bacteria.

Phagocytosis was measured by the ability of *Dictyostelium* cells to reduce the turbidity of a bacterial suspension over time. Although RGBARG disruption had no effect on clearance of *Klebsiella aerogenes*, engulfment of *Escherichia coli* was substantially reduced (Figures 7A and 7B). Therefore, although loss of RGBARG had no effect on engulfing 1 µm beads and is beneficial for uptake of large beads and yeast, it causes a species-specific defect in phagocytosis of bacteria.

The most obvious physical difference between *K. aerogenes* and *E. coli* is their shape (Figures 7C and 7D). Both have similar short axes but *K. aerogenes* average 3.2 µm long compare to an

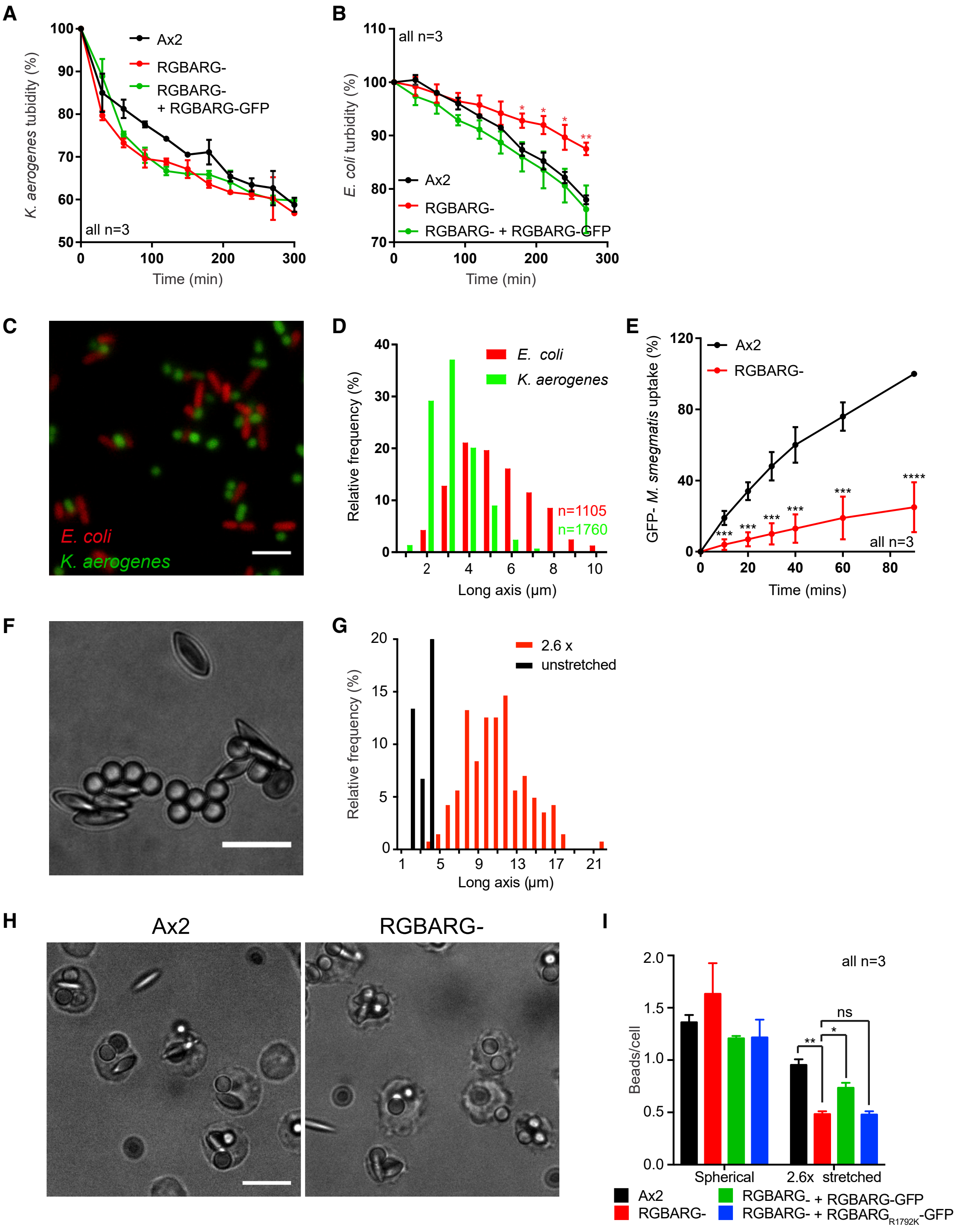

Figure 7. RGBARG– Cells Have Shape-Dependent Phagocytic Defects

(A and B) Phagocytosis of *K. aerogenes* (A) or *E. coli* (B) measured by the decreasing turbidity after addition of *Dictyostelium*.

(C and D) Fluorescence microscopy of GFP-expressing *K. aerogenes* mixed with RFP-expressing *E. coli* (C) demonstrating their different shape and size, quantified in (D).

(E) Phagocytosis of GFP-*M. smegmatis*, measured by flow cytometry.

(F) Brightfield image of a 50:50 mix of untreated and 2.6-fold stretched polystyrene beads.

(G) Quantification of the long axis of stretched beads.

(H) Brightfield images of Ax2 and RGBARG− cells incubated with a mix of stretched and unstretched beads for 30 min.

(I) Quantification of engulfed spherical versus ellipsoid beads within each cell.

Error bars denote standard deviation. *$p < 0.05$, **$p < 0.01$, ***$p < 0.005$ Mann-Whitney t Test. Scale bars, 10 μm.

CellPress
OPEN ACCESS

average long axis of 5.4 μm for *E. coli*. Previous work investigating phagocytosis of different shaped beads by macrophages concluded that elongated shapes are more difficult to engulf [47]. We therefore measured the ability of RGBARG− cells to engulf an additional rod-shaped bacterium (GFP-expressing *Mycobacterium smegmatis,* 3–5 μm long) by flow cytometry. This was again reduced by 75% (Figure 7E), correlating with an inability to phagocytose elongated targets.

The data above are consistent with a role for RGBARG in enabling engulfment of elongated bacteria. However, different bacteria also differ in other aspects such as surface composition, phagocytic receptor activation, and stiffness. To directly test the importance of RGBARG in engulfing different shapes we therefore stretched 3μm latex beads to generate oblate ellipsoids of conserved volume and surface chemistry (Figures 7F and 7G) [48].

To measure relative phagocytosis in the same experiment, cells were incubated with a 1:1 mix of spherical and stretched beads (2.6x aspect ratio) and the number of engulfed beads of each shape quantified by microscopy. Both Ax2 and RGBARG− cells engulfed 3 μm spheres with similar efficiency, but while uptake of ellipsoids was only reduced by 30% in Ax2 cells, it was reduced by 70% in RGBARG mutants (Figures 7H and 7I). These effects were again rescued by re-expression of RGBARG-GFP but not $RGBARG_{R1792K}$-GFP, demonstrating a key role for RGBARG and its RasGAP activity in mediating phagocytosis of elongated particles.

DISCUSSION

In this study, we identify a new component that organizes protrusions into the 3D cup shapes required to engulf extracellular fluid or particles. Consistent with previous work, our data support a model whereby cup formation is guided by the formation of a protrusive rim encircling a static interior domain [8]. We show that RGBARG provides a direct link between the Ras and Rac activities that underlie these different functional domains, providing a mechanism to coordinate cup organization in space and time.

RGBARG is not the only RasGAP in *Dictyostelium* involved in macropinosome formation, but it is the only one to also possess a RhoGEF domain and is therefore unique in its ability to integrate the activities of both GTPase families. No human proteins have an identical domain structure to RGBARG, and although most classical RasGAPs are found in multidomain proteins, none also contain a RhoGEF domain [39]. A screen for RhoGAPs involved in macrophage phagosome formation identified three proteins (ARHGAP12, ARHGAP25, and SH3BP1). Although these all contain PIP_3 binding (PH) or BAR domains, none contain domains linking to other GTPase families [23]. The oncogene TIAM1 contains both RhoGEF and Ras-binding domains, however, and BAR domains are found in conjunction with GAPs or GEFs in several other proteins. Therefore, although mammalian cells also need to coordinate Ras and Rac, this is likely achieved via multiple proteins or a complex.

Multiple GAPs and GEFs collaborate to shape protrusions into cups. This is apparent in the different roles played by RGBARG and NF1; both negatively regulate Ras and are present at the cup interior, and while RGBARG is enriched at the rim, NF1 appears to be uniform throughout the cup [37]. Although there may be some overlapping function at the cup base, RGBARG and NF1 play different roles as NF1 disruption increases cup size and the volume of fluid taken up whereas RGBARG appears more important for cup structure and shape.

This model is doubtless overly simplistic, and other RasGAPs also contribute to shaping active Ras dynamics. For example, the IQGAP-related protein IqgC was also recently shown to have RasGAP activity and localize throughout the interior of macropinocytic and phagocytic cups in *Dictyostelium* [49]. In contrast to NF1 and RGBARG, however, IqgC is reported to be a specific GAP for RasG. As the different Ras isoforms are non-redundant [45], IqgC adds a further layer of complexity to shape engulfment dynamics.

While Ras regulation is becoming clearer, how protrusion is regulated during engulfment is less well understood. Several mammalian studies indicate actin dynamics and protrusion are regulated by the combined activities of Rac1 and CDC42 [23, 31, 33]. Rac1 and CDC42 are differentially activated with active Rac1 throughout the cup and CDC42 activation earlier and more restricted to the rim [25]. We find RGBARG specifically interacts with the atypical Rac isoforms RacG and RacH. Although *Dictyostelium* does not possess a direct CDC42 ortholog, RacG is the most similar protein in sequence, and may therefore be functionally orthologous. No effectors of either RacG or RacH are known, but RacG does not interact with the Rac-binding domain of PAK commonly used as a probe for active Rac1 indicating at least partly distinct effectors [42, 50]. Nonetheless, in cell-free assays, RacG can induce actin nucleation and/or polymerization via the ARP2/3 complex [42] and could therefore at least partly define the protrusive rim, possibly in collaboration with active Rac1.

Whereas constitutively active Rac1 induces the formation of lamellipodial-type protrusions [30], constitutively active RacG and CDC42 both induce filopodia [42, 51]. Recently, it was also shown that filopodial “tent poles” can also drive macropinocytosis in macrophages [52]. It is still not clear whether filopodia and lamellipodial sheets work together to form cups or represent distinct ends of a spectrum of macropinocytic mechanisms, but the involvement of RacG and CDC42 may indicate a conserved mechanism.

How cells restrict protrusion to the periphery of a static interior remains a major unanswered question. It is not yet clear whether the RGBARG GEF domain is active, but simultaneous comparison of Rac and Ras activity clearly shows RGBARG can separate their signaling domains (Figures 3H–3G). If Rac-driven actin polymerization is suppressed by Ras or PIP_3, RGBARG-driven expansion of Rac activation just beyond active Ras provides a plausible mechanism to define the protrusive ring.

The multi-layered regulation of small GTPases is particularly important when cells are challenged to engulf particles or microbes of different shapes. This is critical for amoebae to feed on diverse bacteria or immune cells to capture and kill a wide range of pathogens, but how cells adapt to different target geometries is very poorly understood [47, 53]. To our knowledge, RGBARG− cells are the first mutants reported to have a geometry-specific phagocytic defect, underlining the importance of coordinated Ras and Rac activities. This again differs from the role of NF1, as NF1-deficient Ax2 cells can efficiently engulf

and grow on a wide range of bacteria, including *E. coli* [54]. It is still not known how other regulatory elements or cytoskeletal components adapt to differing shapes, but it is likely that large-scale rearrangements are necessary to accommodate different targets.

In summary, we describe a mechanism to coordinate the activity of Rac and Ras GTPases during engulfment in *Dictyostelium*. The proteins that mediate this coordination in mammalian cells remain unknown. However, we propose a general model by which spatial signals from multiple small GTPases are integrated to shape macropinocytic and phagocytic cups, enabling cells to engulf diverse targets.

STAR★METHODS

Detailed methods are provided in the online version of this paper and include the following:

- KEY RESOURCES TABLE
- RESOURCE AVAILABILITY
  - Lead Contact
  - Materials Availability
  - Data and Code Availability
- EXPERIMENTAL MODEL AND SUBJECT DETAILS
  - *Dictyostelium* culture and molecular biology
- METHOD DETAILS
  - Macropinocytosis assays
  - Phagocytosis assays
  - Microscopy and image analysis
  - Western blotting and lipid overlay assays
  - GAP and GEF biochemistry
  - Ellipsoid bead generation and phagocytosis
  - Chemotaxis assays
- QUANTIFICATION AND STATISTICAL ANALYSIS

SUPPLEMENTAL INFORMATION

Supplemental Information can be found online at https://doi.org/10.1016/j.cub.2020.05.049.

ACKNOWLEDGMENTS

The authors are very grateful to Francisco Rivero for providing the RacG mutant cell line and plasmids, Andrew Peden for the anti-GFP antibody, Gareth Bloomfield and Rob Kay for DdB strains and GFP-RBD constructs, Iwan Evans for the RFP-*E. coli* strain, and Peggy Paschke for generously providing unpublished GFP-reporter plasmids. J.S.K. and J.H.V. are supported by Royal Society University Research Fellowship UF140624. B.A.P. is funded by a BBSRC White Rose PhD studentship (BB/J014443/1) and C.J.M. by an MRC-Discovery Medicine North (DiMEN) studentship (MR/N013840/1). Microscopy studies were supported by UK Medical Research Council grant (G0700091) and Wellcome Trust grant (GR077544AIA). D.T. was supported by MRC core funding to Rob Kay MC_U105115237. A.G. was supported by a Swiss National Science Foundation grant N° 310030_149390 to T.S.

AUTHOR CONTRIBUTIONS

Conceptualization, C.M.B. and J.S.K.; Methodology, C.M.B., B.A.P., A.N., T.S., A.J.P., A.K., and J.S.K.; Investigation, C.M.B., H.P., A.G., J.H.V., C.J.M., B.G., D.T., and J.S.K.; Formal Analysis, C.M.B., A.N., A.K., and J.S.K.; Writing – Original Draft, C.M.B. and J.S.K.; Writing – Review & Editing, all authors; Supervision and Funding Acquisition, T.S., A.J.P., A.K., and J.S.K.

DECLARATION OF INTERESTS

The authors declare no competing interests.

Received: October 25, 2019
Revised: April 20, 2020
Accepted: May 14, 2020
Published: June 11, 2020

REFERENCES

1. Norbury, C.C., Hewlett, L.J., Prescott, A.R., Shastri, N., and Watts, C. (1995). Class I MHC presentation of exogenous soluble antigen via macropinocytosis in bone marrow macrophages. Immunity *3*, 783–791.
2. Sallusto, F., Cella, M., Danieli, C., and Lanzavecchia, A. (1995). Dendritic cells use macropinocytosis and the mannose receptor to concentrate macromolecules in the major histocompatibility complex class II compartment: downregulation by cytokines and bacterial products. J. Exp. Med. *182*, 389–400.
3. Swanson, J.A., and King, J.S. (2019). The breadth of macropinocytosis research. Philos. Trans. R. Soc. Lond. B Biol. Sci. *374*, 20180146.
4. Bloomfield, G., and Kay, R.R. (2016). Uses and abuses of macropinocytosis. J. Cell Sci. *129*, 2697–2705.
5. Commisso, C., Davidson, S.M., Soydaner-Azeloglu, R.G., Parker, S.J., Kamphorst, J.J., Hackett, S., Grabocka, E., Nofal, M., Drebin, J.A., Thompson, C.B., et al. (2013). Macropinocytosis of protein is an amino acid supply route in Ras-transformed cells. Nature *497*, 633–637.
6. Hacker, U., Albrecht, R., and Maniak, M. (1997). Fluid-phase uptake by macropinocytosis in Dictyostelium. J. Cell Sci. *110*, 105–112.
7. Kaplan, G. (1977). Differences in the mode of phagocytosis with Fc and C3 receptors in macrophages. Scand. J. Immunol. *6*, 797–807.
8. Veltman, D.M., Williams, T.D., Bloomfield, G., Chen, B.C., Betzig, E., Insall, R.H., and Kay, R.R. (2016). A plasma membrane template for macropinocytic cups. eLife *5*, e20085.
9. Buckley, C.M., and King, J.S. (2017). Drinking problems: mechanisms of macropinosome formation and maturation. FEBS J. *284*, 3778–3790.
10. Swanson, J.A. (2008). Shaping cups into phagosomes and macropinosomes. Nat. Rev. Mol. Cell Biol. *9*, 639–649.
11. King, J.S., and Kay, R.R. (2019). The origins and evolution of macropinocytosis. Philos. Trans. R. Soc. Lond. B Biol. Sci. *374*, 20180158.
12. Tollis, S., Dart, A.E., Tzircotis, G., and Endres, R.G. (2010). The zipper mechanism in phagocytosis: energetic requirements and variability in phagocytic cup shape. BMC Syst. Biol. *4*, 149.
13. Griffin, F.M., Jr., Griffin, J.A., Leider, J.E., and Silverstein, S.C. (1975). Studies on the mechanism of phagocytosis. I. Requirements for circumferential attachment of particle-bound ligands to specific receptors on the macrophage plasma membrane. J. Exp. Med. *142*, 1263–1282.
14. Williams, T.D., Peak-Chew, S.Y., Paschke, P., and Kay, R.R. (2019). Akt and SGK protein kinases are required for efficient feeding by macropinocytosis. J Cell Sci. *132*, jcs.224998.
15. Marshall, J.G., Booth, J.W., Stambolic, V., Mak, T., Balla, T., Schreiber, A.D., Meyer, T., and Grinstein, S. (2001). Restricted accumulation of phosphatidylinositol 3-kinase products in a plasmalemmal subdomain during Fc gamma receptor-mediated phagocytosis. J. Cell Biol. *153*, 1369–1380.
16. Vieira, O.V., Botelho, R.J., Rameh, L., Brachmann, S.M., Matsuo, T., Davidson, H.W., Schreiber, A., Backer, J.M., Cantley, L.C., and Grinstein, S. (2001). Distinct roles of class I and class III phosphatidylinositol 3-kinases in phagosome formation and maturation. J. Cell Biol. *155*, 19–25.
17. Araki, N., Egami, Y., Watanabe, Y., and Hatae, T. (2007). Phosphoinositide metabolism during membrane ruffling and macropinosome formation in EGF-stimulated A431 cells. Exp. Cell Res. *313*, 1496–1507.

18. Bar-Sagi, D., and Feramisco, J.R. (1986). Induction of membrane ruffling and fluid-phase pinocytosis in quiescent fibroblasts by ras proteins. Science *233*, 1061–1068.
19. Veltman, D.M., Lemieux, M.G., Knecht, D.A., and Insall, R.H. (2014). PIP_3-dependent macropinocytosis is incompatible with chemotaxis. J. Cell Biol. *204*, 497–505.
20. Hoeller, O., Bolourani, P., Clark, J., Stephens, L.R., Hawkins, P.T., Weiner, O.D., Weeks, G., and Kay, R.R. (2013). Two distinct functions for PI3-kinases in macropinocytosis. J. Cell Sci. *126*, 4296–4307.
21. Amyere, M., Payrastre, B., Krause, U., Van Der Smissen, P., Veithen, A., and Courtoy, P.J. (2000). Constitutive macropinocytosis in oncogene-transformed fibroblasts depends on sequential permanent activation of phosphoinositide 3-kinase and phospholipase C. Mol. Biol. Cell *11*, 3453–3467.
22. Araki, N., Johnson, M.T., and Swanson, J.A. (1996). A role for phosphoinositide 3-kinase in the completion of macropinocytosis and phagocytosis by macrophages. J. Cell Biol. *135*, 1249–1260.
23. Schlam, D., Bagshaw, R.D., Freeman, S.A., Collins, R.F., Pawson, T., Fairn, G.D., and Grinstein, S. (2015). Phosphoinositide 3-kinase enables phagocytosis of large particles by terminating actin assembly through Rac/Cdc42 GTPase-activating proteins. Nat. Commun. *6*, 8623.
24. Cox, D., Tseng, C.C., Bjekic, G., and Greenberg, S. (1999). A requirement for phosphatidylinositol 3-kinase in pseudopod extension. J. Biol. Chem. *274*, 1240–1247.
25. Hoppe, A.D., and Swanson, J.A. (2004). Cdc42, Rac1, and Rac2 display distinct patterns of activation during phagocytosis. Mol. Biol. Cell *15*, 3509–3519.
26. Eden, S., Rohatgi, R., Podtelejnikov, A.V., Mann, M., and Kirschner, M.W. (2002). Mechanism of regulation of WAVE1-induced actin nucleation by Rac1 and Nck. Nature *418*, 790–793.
27. Machesky, L.M., and Insall, R.H. (1998). Scar1 and the related Wiskott-Aldrich syndrome protein, WASP, regulate the actin cytoskeleton through the Arp2/3 complex. Curr. Biol. *8*, 1347–1356.
28. West, M.A., Prescott, A.R., Eskelinen, E.L., Ridley, A.J., and Watts, C. (2000). Rac is required for constitutive macropinocytosis by dendritic cells but does not control its downregulation. Curr. Biol. *10*, 839–848.
29. Fujii, M., Kawai, K., Egami, Y., and Araki, N. (2013). Dissecting the roles of Rac1 activation and deactivation in macropinocytosis using microscopic photo-manipulation. Sci. Rep. *3*, 2385.
30. Dumontier, M., Höcht, P., Mintert, U., and Faix, J. (2000). Rac1 GTPases control filopodia formation, cell motility, endocytosis, cytokinesis and development in Dictyostelium. J. Cell Sci. *113*, 2253–2265.
31. Cox, D., Chang, P., Zhang, Q., Reddy, P.G., Bokoch, G.M., and Greenberg, S. (1997). Requirements for both Rac1 and Cdc42 in membrane ruffling and phagocytosis in leukocytes. J. Exp. Med. *186*, 1487–1494.
32. Caron, E., and Hall, A. (1998). Identification of two distinct mechanisms of phagocytosis controlled by different Rho GTPases. Science *282*, 1717–1721.
33. Massol, P., Montcourrier, P., Guillemot, J.C., and Chavrier, P. (1998). Fc receptor-mediated phagocytosis requires CDC42 and Rac1. EMBO J. *17*, 6219–6229.
34. Peter, B.J., Kent, H.M., Mills, I.G., Vallis, Y., Butler, P.J., Evans, P.R., and McMahon, H.T. (2004). BAR domains as sensors of membrane curvature: the amphiphysin BAR structure. Science *303*, 495–499.
35. Dawson, J.C., Legg, J.A., and Machesky, L.M. (2006). Bar domain proteins: a role in tubulation, scission and actin assembly in clathrin-mediated endocytosis. Trends Cell Biol. *16*, 493–498.
36. Dormann, D., Weijer, G., Dowler, S., and Weijer, C.J. (2004). In vivo analysis of 3-phosphoinositide dynamics during Dictyostelium phagocytosis and chemotaxis. J. Cell Sci. *117*, 6497–6509.
37. Bloomfield, G., Traynor, D., Sander, S.P., Veltman, D.M., Pachebat, J.A., and Kay, R.R. (2015). Neurofibromin controls macropinocytosis and phagocytosis in Dictyostelium. eLife *4*, e04940.
38. Williams, T.D., and Kay, R.R. (2018). The physiological regulation of macropinocytosis during Dictyostelium growth and development. J Cell Sci. *131*, jcs.213736.
39. Bos, J.L., Rehmann, H., and Wittinghofer, A. (2007). GEFs and GAPs: critical elements in the control of small G proteins. Cell *129*, 865–877.
40. Vlahou, G., and Rivero, F. (2006). Rho GTPase signaling in Dictyostelium discoideum: insights from the genome. Eur. J. Cell Biol. *85*, 947–959.
41. Somesh, B.P., Neffgen, C., Iijima, M., Devreotes, P., and Rivero, F. (2006). Dictyostelium RacH regulates endocytic vesicular trafficking and is required for localization of vacuolin. Traffic *7*, 1194–1212.
42. Somesh, B.P., Vlahou, G., Iijima, M., Insall, R.H., Devreotes, P., and Rivero, F. (2006). RacG regulates morphology, phagocytosis, and chemotaxis. Eukaryot. Cell *5*, 1648–1663.
43. Chubb, J.R., Wilkins, A., Thomas, G.M., and Insall, R.H. (2000). The Dictyostelium RasS protein is required for macropinocytosis, phagocytosis and the control of cell movement. J. Cell Sci. *113*, 709–719.
44. Junemann, A., Filić, V., Winterhoff, M., Nordholz, B., Litschko, C., Schwellenbach, H., Stephan, T., Weber, I., and Faix, J. (2016). A Diaphanous-related formin links Ras signaling directly to actin assembly in macropinocytosis and phagocytosis. Proc. Natl. Acad. Sci. USA *113*, E7464–E7473.
45. Khosla, M., Spiegelman, G.B., Insall, R., and Weeks, G. (2000). Functional overlap of the dictyostelium RasG, RasD and RasB proteins. J. Cell Sci. *113*, 1427–1434.
46. Inaba, H., Yoda, K., and Adachi, H. (2017). The F-actin-binding RapGEF GflB is required for efficient macropinocytosis in *Dictyostelium*. J. Cell Sci. *130*, 3158–3172.
47. Champion, J.A., and Mitragotri, S. (2006). Role of target geometry in phagocytosis. Proc. Natl. Acad. Sci. USA *103*, 4930–4934.
48. Ho, C.C., Keller, A., Odell, J.A., and Ottewill, R.H. (1993). Preparation of Monodisperse Ellipsoidal Polystyrene Particles. Colloid Polym. Sci. *271*, 469–479.
49. Marinović, M., Mijanović, L., Šoštar, M., Vizovišek, M., Junemann, A., Fonović, M., Turk, B., Weber, I., Faix, J., and Filić, V. (2019). IQGAP-related protein IqgC suppresses Ras signaling during large-scale endocytosis. Proc. Natl. Acad. Sci. USA *116*, 1289–1298.
50. Park, K.C., Rivero, F., Meili, R., Lee, S., Apone, F., and Firtel, R.A. (2004). Rac regulation of chemotaxis and morphogenesis in Dictyostelium. EMBO J. *23*, 4177–4189.
51. Nobes, C.D., and Hall, A. (1995). Rho, rac, and cdc42 GTPases regulate the assembly of multimolecular focal complexes associated with actin stress fibers, lamellipodia, and filopodia. Cell *81*, 53–62.
52. Condon, N.D., Heddleston, J.M., Chew, T.L., Luo, L., McPherson, P.S., Ioannou, M.S., Hodgson, L., Stow, J.L., and Wall, A.A. (2018). Macropinosome formation by tent pole ruffling in macrophages. J Cell Biol., jcb.201804137.
53. Champion, J.A., and Mitragotri, S. (2009). Shape induced inhibition of phagocytosis of polymer particles. Pharm. Res. *26*, 244–249.
54. Buckley, C.M., Heath, V, L., Gueho, A., Bosmani, C., Knobloch, P., Sikakana, P., Personnic, N., Dove, S.K., Michell, R.H., Meier, R., et al. (2019). PIKfyve/Fab1 is required for efficient V-ATPase and hydrolase delivery to phagosomes, phagosomal killing, and restriction of Legionella infection. PloS pathogens. *15*, e1007551.
55. Hagedorn, M., and Soldati, T. (2007). Flotillin and RacH modulate the intracellular immunity of Dictyostelium to Mycobacterium marinum infection. Cell. Microbiol. *9*, 2716–2733.
56. Benghezal, M., Fauvarque, M.O., Tournebize, R., Froquet, R., Marchetti, A., Bergeret, E., Lardy, B., Klein, G., Sansonetti, P., Charette, S.J., and Cosson, P. (2006). Specific host genes required for the killing of Klebsiella bacteria by phagocytes. Cell. Microbiol. *8*, 139–148.
57. Gotthardt, D., Warnatz, H.J., Henschel, O., Brückert, F., Schleicher, M., and Soldati, T. (2002). High-resolution dissection of phagosome maturation reveals distinct membrane trafficking phases. Mol. Biol. Cell *13*, 3508–3520.

58. Watts, D.J., and Ashworth, J.M. (1970). Growth of myxameobae of the cellular slime mould Dictyostelium discoideum in axenic culture. Biochem. J. *119*, 171–174.

59. Paschke, P., Knecht, D.A., Silale, A., Traynor, D., Williams, T.D., Thomason, P.A., Insall, R.H., Chubb, J.R., Kay, R.R., and Veltman, D.M. (2018). Rapid and efficient genetic engineering of both wild type and axenic strains of Dictyostelium discoideum. PLoS ONE *13*, e0196809.

60. Schindelin, J., Arganda-Carreras, I., Frise, E., Kaynig, V., Longair, M., Pietzsch, T., Preibisch, S., Rueden, C., Saalfeld, S., Schmid, B., et al. (2012). Fiji: an open-source platform for biological-image analysis. Nat. Methods *9*, 676–682.

61. Bloomfield, G., Tanaka, Y., Skelton, J., Ivens, A., and Kay, R.R. (2008). Widespread duplications in the genomes of laboratory stocks of Dictyostelium discoideum. Genome Biol. *9*, R75.

62. Fey, P., Dodson, R.J., Basu, S., and Chisholm, R.L. (2013). One stop shop for everything Dictyostelium: dictyBase and the Dicty Stock Center in 2012. Methods Mol. Biol. *983*, 59–92.

63. Veltman, D.M., Akar, G., Bosgraaf, L., and Van Haastert, P.J. (2009). A new set of small, extrachromosomal expression vectors for Dictyostelium discoideum. Plasmid *61*, 110–118.

64. Williams, T., and Kay, R.R. (2018). High-throughput Measurement of Dictyostelium discoideum Macropinocytosis by Flow Cytometry. J. Vis. Exp. (139) https://doi.org/10.3791/58434.

65. Sattler, N., Monroy, R., and Soldati, T. (2013). Quantitative analysis of phagocytosis and phagosome maturation. Methods Mol. Biol. *983*, 383–402.

66. Rivero, F., and Maniak, M. (2006). Quantitative and microscopic methods for studying the endocytic pathway. Methods Mol. Biol. *346*, 423–438.

67. Laevsky, G., and Knecht, D.A. (2001). Under-agarose folate chemotaxis of Dictyostelium discoideum amoebae in permissive and mechanically inhibited conditions. Biotechniques *31*, 1140–1142, 1144, 1146–1149.

CellPress
OPEN ACCESS

STAR★METHODS

KEY RESOURCES TABLE

REAGENT or RESOURCE		SOURCE	IDENTIFIER
Antibodies			
Anti-GFP Rabbit polyclonal		Andrew Peden, University of Sheffield	N/A
Alexa680-labeled streptavidin		Life Technologies	Cat#S21378
Anti-GFP antibody		Santa Cruz Biotechnology	Cat#SC9996; RRID: AB_627695
Bacterial and Virus Strains			
GFP expressing *M. smegmatis*		Thierry Soldati Laboratory, University of Geneva [55].	N/A
RFP-*E.coli* DH5α		Iwan Evans Laboratory, University of Sheffield	N/A
GFP-expressing *K. aerogenes*		Pierre Cosson Laboratory, University of Geneva [56].	N/A
E. coli Rosetta cells		Novagen	Cat#70954
Biological Samples			
TRITC-labeled *S. cerevisiae* RH210-1B		Thierry Soldati Laboratory, University of Geneva [57].	N/A
S. cerevisiae MATa ura3-52 leu2-3,112 his3200 trp1-1 lys2-801		Kathryn Ayscough Laboratory, University of Sheffield	Ayscough Lab strain ID: KAY389
Chemicals, Peptides, and Recombinant Proteins			
70kDa FITC Dextran		Sigma-Aldrich	Cat#46945
1 μm YG-carboxylated polystyrene beads		Polysciences	Cat#15702-10
4.5 μm YG-carboxylated polystyrene beads		Polysciences	Cat#16592-5
3 μm non-functionalised polybead microspheres		Polysciences	Cat#17134-15
PIP strips		Echelon Biosciences	Cat#P-6001
PIP Array		Echelon Biosciences	Cat# P-6100
His-NF1 GAP domain (AA 2530-3158)		This work	N/A
His-RGBAR GAP domain (AA 1717-2045)		This work	N/A
Glutathione Sepharose beads		GE Healthcare	Cat# 17075601
HisTrap excel-affinity column		GE Healthcare	Cat# 17371205
Maltose Binding protein trap affinity column		GE Healthcare	Cat# 28918778
HiPrep 16/60 Sephacryl columns		GE Healthcare	Cat# 17116501
Experimental Models: Cell Lines			
Strain	Parent	Source	Identifier (Dictybase reference)
Genetically clean wild-type subclone of NC4	NC4	Kay group (MRC-LMB)	DdB (DBS0350772)
Axenic mutant of DdB containing *axeB* (Neurofibromin, NF1) mutation.	DdB	Kay group (MRC-LMB). Originally from [58]	MRC-Ax2 (DBS0235521)
rgbA/DDB_G0269934 knockout (using pCB43)	MRC-Ax2	This work	JSK02
rgbA/DDB_G0269934 knockout (Blasticidin)	MRC-Ax2	This work	JSK03
Random integrant of pCB43	MRC-Ax2	This work	JSK04
Devreotes group Ax2 strain	DdB	[42]	Ax2D (DBS0350907)
racG knockout	Ax2D	[42]	RacG- (DBS0236849)
axeB (Neurofibromin, NF1) knockout	DdB	[37]	HM1591 (DBS0350773)
rgbA/DDB_G0269934 knockout (using pCB62)	DdB	This work	JSK18
rgbA/DDB_G0269934 knockout (using pCB62)	DdB	This work	JSK19
Oligonucleotides			
RGBARG KO cassette 5′ arm fw primer: CCACCAATCAATACTAGTTCAGGT		This work	N/A

(*Continued on next page*)

Continued

REAGENT or RESOURCE	SOURCE	IDENTIFIER
RGBARG KO cassette 5′ arm rv primer: gatagctctgcctactgaagCCAATGGTTCAGG TTTACTTGG	This work	N/A
RGBARG KO cassette 3′ arm fw primer: ctactggagtatccaagctgGCTCCTTCTCCAT TGGTATTGG	This work	N/A
RGBARG KO cassette 3′ arm rv primer: CCAATGATGAAACGATTGACTGG	This work	N/A
RGBARG KO screening fw primer: GGTAATGTTATAAATAGGCCACAACC	This work	N/A
RGBARG KO screening rv primer: gctagcTTTATACATTGAAGATGGATCACCTAAG	This work	N/A
Recombinant DNA		
Extrachromosomal N-terminal GFP-fusion expression	Douwe Veltman, unpublished	pDM1043
Extrachromosomal C-terminal GFP-fusion expression	Douwe Veltman, unpublished	pDM1045
Extrachromosomal C-terminal RFP fusion expression	Douwe Veltman, unpublished	pDM1097
loxP-Blasticidin selection cassette	[59]	pDM1079
loxP-G418 selection cassette	[59]	pDM1082
Ras binding domain from Raf1. Active Ras reporter	[37]	GFP-RBD
PH_{CRAC}-GFP expression vector	[19]	pDM631
PH_{CRAC}-mCherry expression in pDM1097	Douwe Veltman, unpublished	pDM1142
RGBARG-GFP expression vector	This work	pCB34
RGBARGΔRCC1-GFP expression vector	This work	pCB83
RGBARGΔGEF-GFP expression vector	This work	pCB71
RGBARGΔBAR-GFP expression vector	This work	pCB72
RGBARGΔGAP-GFP expression vector	This work	pCB73
$RGBARG_{R1792K}$-GFP expression vector	This work	pCB122
RCC1(RGBARG)-GFP expression vector	This work	pCB112
BAR(RGBARG)-GFP expression vector	This work	pCB114
GAP(RGBARG)-GFP expression vector	This work	pCB115
rgbA- knockout vector (Blasticidin selection, pDM1079)	This work	pCB43
rgbA- knockout vector (G418 selection, pDM1082)	This work	pCB62
GFP-myoIB/PH_{PkgE}-mCherry co-expression vector	Peggy Paschke, unpublished	pPI84
PH_{PkgE}-GFP/PTEN-mCherry co-expression vector	Peggy Paschke, unpublished	pPI356
GFP-RBD/$PakB_{CRIB}$-mCherry active Ras/Rac reporter co-expression vector	Peggy Paschke, unpublished	pPI587
HSPC300-GFP (SCAR complex reporter) expression vector	Douwe Veltman, unpublished	pDM1091
Software and Algorithms		
Graphpad Prism (v7)	GraphPad Software	www.graphpad.com
Volocity (v6.3)	Perkin Elmer	N/A
Zen black	Zeiss	https://www.zeiss.com/microscopy/int/products/microscope-software/zen.html
ImageJ (v1.52)	Fiji [60]	https://imagej.nih.gov/ij/
Igor pro 8	WaveMetrics	https://www.wavemetrics.com/
FlowJo (v9)	FlowJo	https://www.flowjo.com/
GraFit (v5.0)	Erithacus Software	http://www.erithacus.com/grafit/

CellPress
OPEN ACCESS

RESOURCE AVAILABILITY

Lead Contact

Further information and requests for resources and reagents should be directed to and will be fulfilled by the Lead Contact, Jason King (jason.king@sheffield.ac.uk).

Materials Availability

All *Dictyostelium* strains and plasmids generated for this work are deposited in, and available from the *Dictyostelium* stock center (www.dictybase.org). Plasmids kindly provided by Dr. Peggy Paschke are all available from Addgene (www.addgene.org).

Data and Code Availability

Code used to calculate average profiles of protein localization across cups can be found on Github: https://github.com/tonza17/RGBARG

EXPERIMENTAL MODEL AND SUBJECT DETAILS

Dictyostelium culture and molecular biology

Unless otherwise stated, axenic *Dictyostelium* strains were derived from the MRC-Ax2 axenic strain (DBS0236849) provided by the Kay laboratory and were routinely cultured in filter sterilized HL-5 medium (Formedium) at 22°C. RacG mutants and corresponding parental strain (from the Devreotes group, Johns Hopkins, Ax2D) were kind gifts from Francisco Rivero (University of Hull) [42]. Growth rates were measured by seeding cells at 0.5 × 10^5/mL in HL-5 and counting cell number twice daily for three days. Growth rate was then calculated by fitting an exponential growth curve using Graphpad Prism software. Cells were transformed by electroporation: 6 × 10^6 cells were resuspended in 0.4 mL of ice-cold E-buffer (10 mM KH_2PO_4 pH 6.1, 50 mM sucrose) and transferred to 2 mm electroporation cuvette containing DNA (0.5 μg for extrachromosomal plasmids, 15 μg for knockout vectors). Cells were then electroporated at 1.2 kV and 3 μF capacitance with a 5 Ω resistor in series using a Bio-Rad Gene Pulser II. After 24 h transformants were selected in either 20 μg/mL hygromycin (Invitrogen), 10 μg/mL G418 (Sigma-Aldrich) or 10 μg/mL blasticidin (Melford).

Nonaxenic mutants were generated from the DdB (Wel) subclone of NC-4 shown to be the lab isolate with fewest duplications and parent strain of Ax2 [61]. The published DdB strain and corresponding NF1 mutants were gifts from Rob Kay (MRC-LMB, Cambridge) [37]. All DdB-derived strains were routinely maintained on lawns of *Klebsiella aerogenes* bacteria in SM agar plates (Formedium) at 22°C. 24 h prior to all experiments, cells were washed free of bacteria and transferred to HL5 medium supplemented with 20% fetal calf serum to axenically adapt [38]. DdB and its derivatives were transformed as described in [59]: 2 × 10^6 cells were resuspended in 100 μl ice-cold H40 buffer (40mM HEPES, 1mM $MgCl_2$ pH 7.0) and 10μg DNA in 2mm cuvettes and exposed to 2 pulses of 400V 5 s apart and 3 μF capacitance in a square-wave electroporator (BTX EMC399, Harvard Apparatus). Cells were then grown in Petri dishes in a suspension of *K. aerogenes* in SorMC buffer (15mM KH_2PO_4, 2mM Na_2HPO_4, 50μM $MgCl_2$, 50 μM $CaCl_2$, pH 6.0), and transformants selected with G418 (Sigma-Aldrich) using either 5 μg/mL for knockouts or 10 μg/mL for extrachromosomal vectors.

BAR domain containing proteins were identified by multiple BLAST searches using Dictybase (www.dictybase.org) [62]. Coding sequences were then amplified by PCR from vegetative Ax2 cDNA adding compatible restriction sites for subcloning into the BglII/SpeI sites of the N- and C-terminal GFP-fusion *Dictyostelium* extrachromosomal expression vectors pDM1043 and pDM1045 (non-axenically selectable versions of the pDM modular expression system (Veltman et al., 2009)). Truncation and point mutants of RGBARG were also generated by PCR and expressed using pDM450 [63]. The *rbgA* (*DDB_G0269934*) knockout construct was generated by PCR fusion of ~1Kb 5′ and 3′ recombination arms with the floxed blasticidin selection cassette from pDM1079, as described in detail in (Paschke et al., 2018). To select bacterially-grown cells, an identical construct was made using the G418 selection cassette from pDM1082. After transformation, independent clones were obtained by dilute plating in 96 well plates. Disruption of the RGBARG locus was screened by PCR from genomic DNA isolated from 1 × 10^6 cells lysed in 100 μl 10mM Tris- HCl pH8.0, 50 mM KCl, 2.5mM $MgCl_2$, 0.45% NP40, 0.45% Tween 20 and 0.4 mg/mL Proteinase K (NEB). After 5 min incubation at room temperature, the proteinase K was denatured at 95°C for 10 min prior to PCR analysis. The Ras binding domain (RBD) of RAF1-GFP construct used as an active Ras reporter was a gift from Gareth Bloomfield [37]. The PTEN-mCherry/PH_{PkgE}-GFP, PH_{PkgE}-RFP/GFP-MyoIB and RBD-GFP/PAK1$_{CRIB}$-GFP co-expression constructs were all gifts from Peggy Paschke (Beatson Institute, Glasgow).

METHOD DETAILS

Macropinocytosis assays

Bulk macropinocytosis was measured by flow cytometry as in [64]. For Ax2 cells and their derivatives, 5 × 10^4 cells were seeded in 50 μl HL5 medium per well of a 96 well plate, with duplicate wells for 0, 5, 10, 30, 60, 90 and 120 min time points and left for 2 h to settle. 50 μl of 1 mg/mL TRITC-dextran (70kDa in HL5; Sigma-Aldrich) was then added to the 120 min point wells, followed by the others at the appropriate time. After the final time point, the media was removed by flicking into the sink, and the plate washed by submerging in a dish of ice-cold KK2 buffer and flicking again. 100 μl of ice-cold KK2 buffer with 5mM NaN_3 was then added to each well on ice for 15 min to resuspend the cells. The plate was then analyzed by flow cytometry (Attune NxT fitted

with a 96-well plate autosampler; Life Technologies) measuring > 5,000 cells per sample. Fluid uptake by DdB-derived cells was measured in the same way, except cells were grown and measured in HL5-medium supplemented with 20% FCS and 5mg/mL TRITC dextran was used to compensate for the lower uptake. Fluid uptake was calculated by the average TRITC fluorescence at each time point after subtracting background (0 min value) and normalized relative to the appropriate parental control at 120 min.

Macropinosome volume was measured by incubating cells for 5 min in 0.1 mg/mL FITC-dextran and obtaining Z stacks on a spinning disc confocal microscope. FITC is pH-sensitive and the sensitivity was set so only new non-acidified macropinosomes were visible as transition to neutral post-lysosomes takes > 30 min. For analysis, individual cells were cropped out, randomized, and volume calculated from manually measuring the maximum diameter of each macropinosome in each cell, assuming they were spherical.

Phagocytosis assays

Phagocytosis of fluorescent beads was measured by flow cytometry as previously described in detail [65]. Briefly, 1 or 4.5 μm diameter YG-carboxylated polystyrene beads (Polysciences Inc) were shaken with 2 × 10^6 *Dictyostelium* /mL at ratios of 200:1 and 10:1 respectively. 500 μl samples were removed at each time point and added to 3 mL ice-cold Sorenson sorbitol buffer (SSB; 15 mM KH_2PO_4, 2 mM Na_2HPO_4, 120 mM Sorbitol) containing 5 mM sodium azide. Samples were then centrifuged at 100 x g for 10 min, pellets resuspended in SSB and analyzed on an Attune NxT flow cytometer (Life Technologies). Analysis was performed using FlowJo software as described [65].

To measure uptake of GFP-expressing *M. smegmatis* by flow cytometry, the bacteria were grown to an OD_{600} of 1 under shaking conditions (150 rpm) at 32°C in Middlebrook 7H9 medium (Difco), 0.2% glycerol and 0.05% Tween 80 supplemented with 10% OADC (Beckton Dickinson). To avoid clumping they were cultivated in presence of 5 mm glass beads. Prior to use *M. smegmatis* were pelleted by centrifugation at 10,625 x *g* for 4 min and resuspended in 1 mL HL5 medium. Bacteria were then unclumped by passing through a 26-guage needle several times, before adding a 1/10th volume of bacteria to a *Dictyostelium* culture and processing as above.

To measure phagocytosis of bacteria by decreasing turbidity, an overnight bacterial culture in LB was diluted 1:25 and grown at 37°C until an OD600 of 0.7 before pelleting and resuspension in SSB at an OD600 of 0.8. This was then added to an equal volume of *Dictyostelium* at 2 × 10^7 cells/mL in SSB at room temperature and shaken in flasks. The OD600 was then measured over time.

Phagocytosis and TRITC labeling of heat killed *S. cerevisiae* was performed essentially as previously described [66]. *Dictyostelium* at 1 × 10^6 cells/mL in HL5 were seeded in glass-bottomed microscopy dishes (Mat-tek) and left for 1 h prior to addition of a 5-fold excess of yeast. After 30 min, the fluorescence of extracellular yeast was quenched by addition of 0.2 mg/mL trypan blue and images of multiple fields of view taken on a wide-field microscope scoring > 100 cells per condition.

Microscopy and image analysis

Live cell imaging was performed in glass-bottomed microscopy dishes (Mat-Tek) with cells seeded the preceding day in filtered HL-5 medium, unless otherwise stated. Spinning disc images were captured using a Perkin-Elmer Ultraview VoX spinning disk microscope running Volocity software with a UplanSApo 60x oil immersion objective (NA 1.4) and Hammamatsu C9100-50-EM0CCD camera. Laser scanning confocal images were obtained using a Zeiss LSM880 Airyscan confocal equipped with a Fastscan detector, and a 63x 1.4 NA objective. Images were acquired in fastscan mode and deconvolved by airyprocessessing using Zen black software (Zeiss).

All image analysis was performed using ImageJ (https://imageJ.nih.gov). For relative Rac and Ras activity profiles, linescans were drawn along the cell membrane from the center of the cup to ~3 μm outside the rim. Line profiles from multiple cups were integrated by manually determining the edge of the Ras patch and defining this point as 0 μm. Fluorescence was then normalized to the average intensity of the first 10 points of the profile, minus background (average intensity along a ~3 μm non-protrusive membrane region).

Average plots of RGBARG protein enrichment across cups generated using a custom script in Igor Pro (Wavemetrics). For this, confocal images were captured and linescans of GFP-fluorescence intensity measured from the protrusive tip to tip. The average signal from a 1-2 μm non-protruding region of the cell was also measured as was the local background outside each cell. Local background was subtracted and signal across the cup divided by the non-protruding membrane signal to give fold-enrichment. To compare enrichment across multiple cups, normalized linescans were extrapolated over 1000 points and averaged. Enrichment at the cup tip was measured by the average of the first 100 points of the profile for each cup.

The bacterial long axis was measured automatically from widefield images of either GFP or RFP expressing bacteria in ImageJ. Individual bacteria were identified by thresholding and long axis measured using the Feret's diameter function.

Western blotting and lipid overlay assays

Western blotting was performed by standard techniques, separating proteins by SDS-PAGE and probing using a custom rabbit polyclonal antibody to GFP (gift from Andrew Peden). Endogenously biotinylated proteins were used as a loading control, using Alexa680-labeled streptavidin (Life Technologies)(Davidson et al., 2013). Blots were imaged Li-Corr odyssey SA fluorescence gel imager.

For lipid overlay assays, 1 × 10^7 Ax2 cells expressing the BAR domain fused to GFP (pCB114) were washed once in SSB, lysed in 600 μl RIPA buffer (50 mM Tris-HCl pH7.5, 150 mM NaCl, 0.1% SDS, 2 mM EDTA, 0.5% sodium deoxycholate, 1 x HALT protease inhibitors (Thermo Fisher), 0.5% Triton X-100) and left on ice for 45 min. Insoluble material was then removed by centrifugation at 15,871 x g for 20 min at 4°C. PIP strips or Arrays (Echelon Biosciences) were blocked in 3% fatty acid-free bovine serum albumin

(BSA) in TBS-T (20 mM Tris base, 150 mM NaCl, 0.05% Tween20, pH7.2). Samples were then diluted in TBS-T and incubated with the strips for 1 h at 22°C, before washing and processing as for western blotting.

GAP and GEF biochemistry

Interactions with recombinant GST-Rac isoforms were performed as described previously (Plak et al., 2013). *Dictyostelium* cells expressing GST-Rac bait proteins and GFP-fused to the GEF domain of RGBARG were expressed and lysed in 2mls of buffer (10 mM Na_2HPO_4 pH7.2, 1% Triton X-100, 10% glycerol, 150 mM NaCl, 10 mM MgCl2, 1 mM EDTA, 1 mM Na_3VO_4, 5 mM NaF) including protease inhibitor cocktail (Roche). Lysates were mixed with glutathione Sepharose beads (GE Healthcare) and incubated overnight at 4°C. Unbound proteins were washed away with PBS, and bound proteins detected by western blot using an anti-GFP antibody (SC9996).

For GAP activity measurements, His-NF1 GAP domain (AA 2530-3158) and MBP-His-RGBARG GAP domain (AA 1717-2045) were produced and isolated from *E. coli* Rosetta cells (Novagen). His-NF1 GAP was purified using a HisTrap excel - affinity column (GE Healthcare) and eluted in buffer containing 50 mM Tris, 50 mM NaCl, 5% Glycerol, 3 mM β-Mercaptoethanol and 200 mM Imidazole, pH7.5. MBP-His-RGBAR GAP, was purified by Maltose Binding Protein Trap (MBP-Trap) - affinity column (GE Healthcare) and eluted in 20 mM Tris, 200 mM NaCl, 5% Glycerol 1 mM β-Mercaptoethanol and 10mM Maltose, pH7.5. Proteins were further purified by size exclusion chromatography (Sephacryl 16/60, GE Healthcare) and stored in 50 mM Tris, 50 mM NaCl, 5 mM DTT, and 5 mM MgCl2, pH7,5.1.

1 μM of the indicated Ras proteins with and without equal amount of indicated GAP domain was incubated with 50 μM of GTP at 20°C in 50 mM Tris pH 7.5, 50 mM NaCl and 5 mM $MgCl_2$. At each time point the GDP content of the samples was analyzed by a HPLC (Thermo Ultimate 3000): a reversed phase C18 column was employed to detect GDP and GTP content (in %) as previously described (Eberth and Ahmadian, 2009). Linear rates of GDP production (first 4-8 time points) were calculated using GraFit 5.0 (Erithacus software).

Ellipsoid bead generation and phagocytosis

3 μm unmodified non-fluorescent polystyrene beads (Polysciences Inc.) were embedded in polyvinyl alcohol (PVA) film and stretched as previously described (Ho et al., 1993). Briefly, 2.8 mL beads of bead solution were added to 20 mL 25% w/w dissolved PVA solution and poured into a 10.5 × 10.5 cm plastic mold to create a film. These were cut into 3 × 2 cm strips, marked with a grid to follow deformation and placed in a custom stretching device as described in detail in (Ho et al., 1993). Films were then placed in a 145°C oil bath to soften beads and film and slowly pulled to the desired length. After cooling, beads were extracted from the central region where the grid was deformed evenly. This part was cut into small pieces and rotated in 10 mls of a 3:7 mix of isopropanol:water overnight at 22°C to dissolve. Beads were aliquoted and twice heated at 75°C for 10 min and washed in isopropanol: water. Beads were then washed twice in isopropanol:water at 22°C, before two washes in water. The amount of stretch was measured by imaging on an inverted microscope and manually measuring their length in ImageJ.

To measure phagocytosis, equal numbers of stretched and unstretched beads were mixed, sonicated and incubated at a 10-fold excess to cells at 1×10^6 /mL, shaking in HL5. After 30 min, 500 μl samples were added to 3 mL SSB with 5 mM sodium azide to detach unengulfed beads. Cells were washed in ice-cold SSB, transferred to a microscopy dish and allowed to adhere for 10 min before imaging and the number of each shape bead internalised quantified manually.

Chemotaxis assays

Chemotaxis assays were performed under agar using a folate chemoattractant as previously described [67]. For each repeat, 8 mL of 1% agarose-HL5 solution was poured into a 60 mm × 15 mm plastic Petri dish, and was left to set overnight in a humidified chamber. Three parallel wells, set 5 mm apart, of 2 mm x 39 mm were cut into the gel using a razor blade. 200 μL of 0.1 mM folate was added to the central well and 200 ìL *Dictyostelium* cells at 5×10^6 cells/mL, were added to the remaining two wells. Plates were returned to the humidifying chamber for a folate gradient to establish. After approximately three h cells performing chemotaxis were imaged using an LD A-plan ×20 air phase objective on a Zeiss Axiovert widefield microscope with a Hamamatsu Orca ER camera, running μManager software (www.micro-manager-org). Images were taken every 30 s for a total of 90 min and compiled into time-lapse Videos. For analysis 10 random cells were manually tracked using the manual tracking plugin for Fiji (ImageJ). Speed was measured as the total path distance divided by time, chemotaxtic index is the distance moved in the direction of the gradient/total path length.

QUANTIFICATION AND STATISTICAL ANALYSIS

Statistical analysis was performed using the software described in the sections above namely GraphPad Prism and Igor Pro. Details of statistical tests used, errors shown and significance values are located in figure legends. Unless otherwise stated n represents the number of independent experiments, and statistical comparisons are between mutant strains and the appropriate parental control. In all figures *** indicates $p < 0.005$, ** $p < 0.01$ and * $p < 0.05$.

Current Biology, Volume *30*

Supplemental Information

Coordinated Ras and Rac Activity Shapes Macropinocytic Cups and Enables Phagocytosis of Geometrically Diverse Bacteria

Catherine M. Buckley, Henderikus Pots, Aurelie Gueho, James H. Vines, Christopher J. Munn, Ben A. Phillips, Bernd Gilsbach, David Traynor, Anton Nikolaev, Thierry Soldati, Andrew J. Parnell, Arjan Kortholt, and Jason S. King

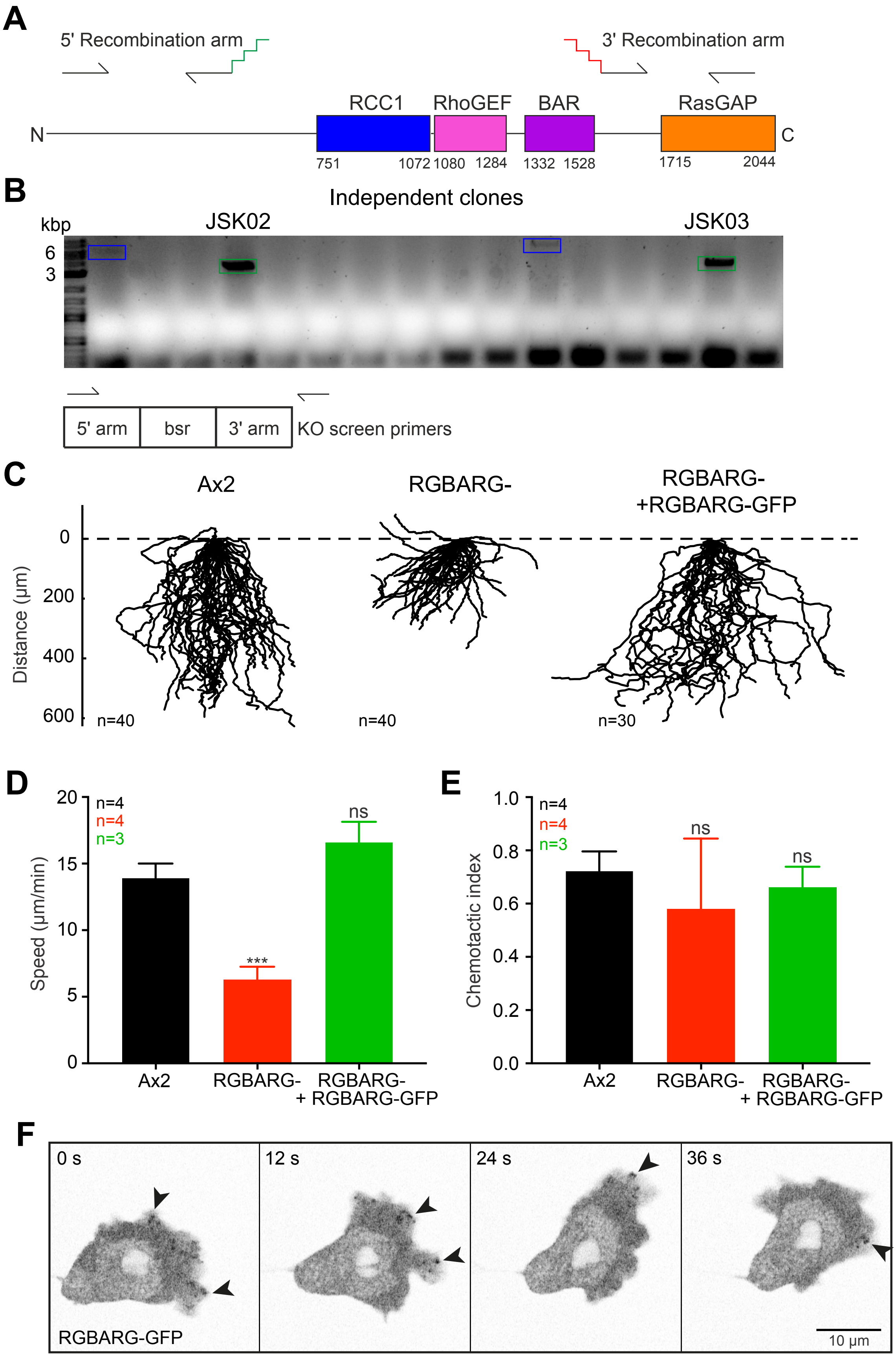

Figure S1: Generation of RGBARG mutants and analysis of chemotaxis, related to Figure 2. (A) Schematic of the *rgbA* genomic locus indicating the position of the regions encoding each domain, and the 5' and 3' recombination arms amplified by PCR. These were attached either side of a blasticidin selection cassette by fusion PCR and used to transform Ax2 cells and delete 3.6 kbp of the gene. (B) PCR screen of transformants, using one primer within the 5'recombination arm and another after the 3' arm. Clones with *rgbA* disrupted will give a product of 3.1 kbp (green box), the wild-type locus is 6.1 kbp (blue boxes). (C) Loss of RGBARG also disrupts chemotaxis. Tracks of Ax2, RGBARG- and RGBARG- cells rescued with RGBARG-GFP chemotaxing towards folate under agar for 45 minute movies. (D) Average cell speed and (E) Chemotactic index (movement up gradient/total pathlength). Bars indicate mean ± SEM. *** P<0.005 Mann-Whitney T-Test. (F) Localisation of RGBARG-GFP in Ax2 cells chemotaxing under agar. Arrows indicate the small, highly transient puncta observed.

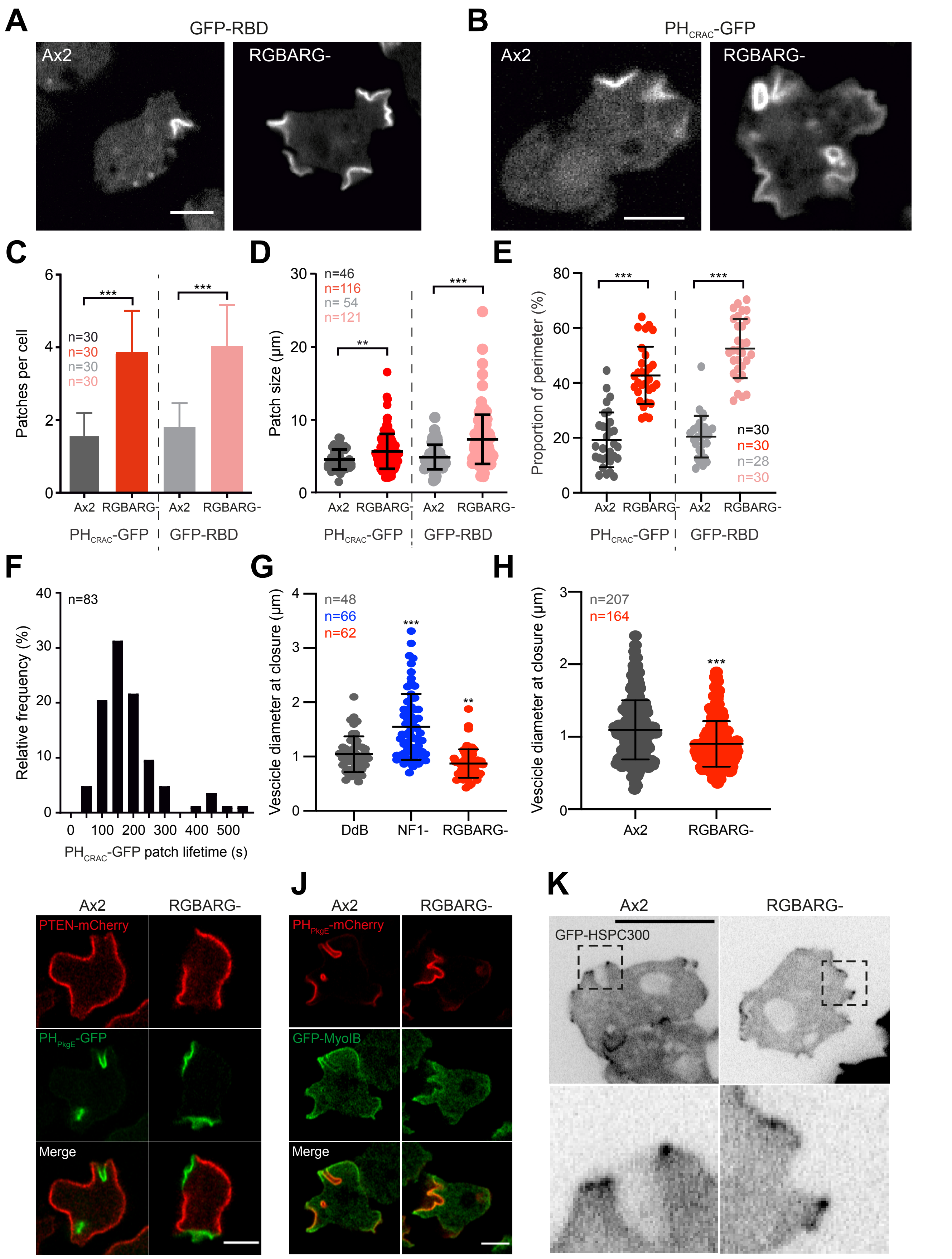

Figure S2: Analysis of cup signalling in Ax2-derived RGBARG mutants, related to Figure 3. (A) Single confocal planes of the active Ras probe GFP-RBD in Ax2 and RGBARG- cells. (B) Equivalent images using PH_{CRAC}-GFP. (C-E) Is quantification of the patch frequency, size and proportion of cell diameter respectively. (F) Histogram of PH_{CRAC}-GFP patch lifetime in Ax2 cells from maximum intensity projection movies (E.g. Movie 6). Lifetime was measured from the first frame an independent patch was visible to when it was completely removed from both the surface and any internalized vesicle. (G) and (H) is quantification of the size of the vesicles formed, as measured from 3D-projection timelapses of cells expressing (G) GFP-RBD or (H) PH_{CRAC}-GFP (Arrows on Figure 3 and see Movies 5 and 6). (I) Localisation of PTEN-mCherry and (J) MyoIB-GFP in Ax2 and RGBARG- cells, relative to forming cups, marked by the PIP_3 probe PHPkgE-GFP. (K) Recruitment of the HSPC300 subunit of the SCAR/WAVE complex to cup protrusions. Bottom panels are enlargements of the boxed regions above. All scale bars = 5 µm.

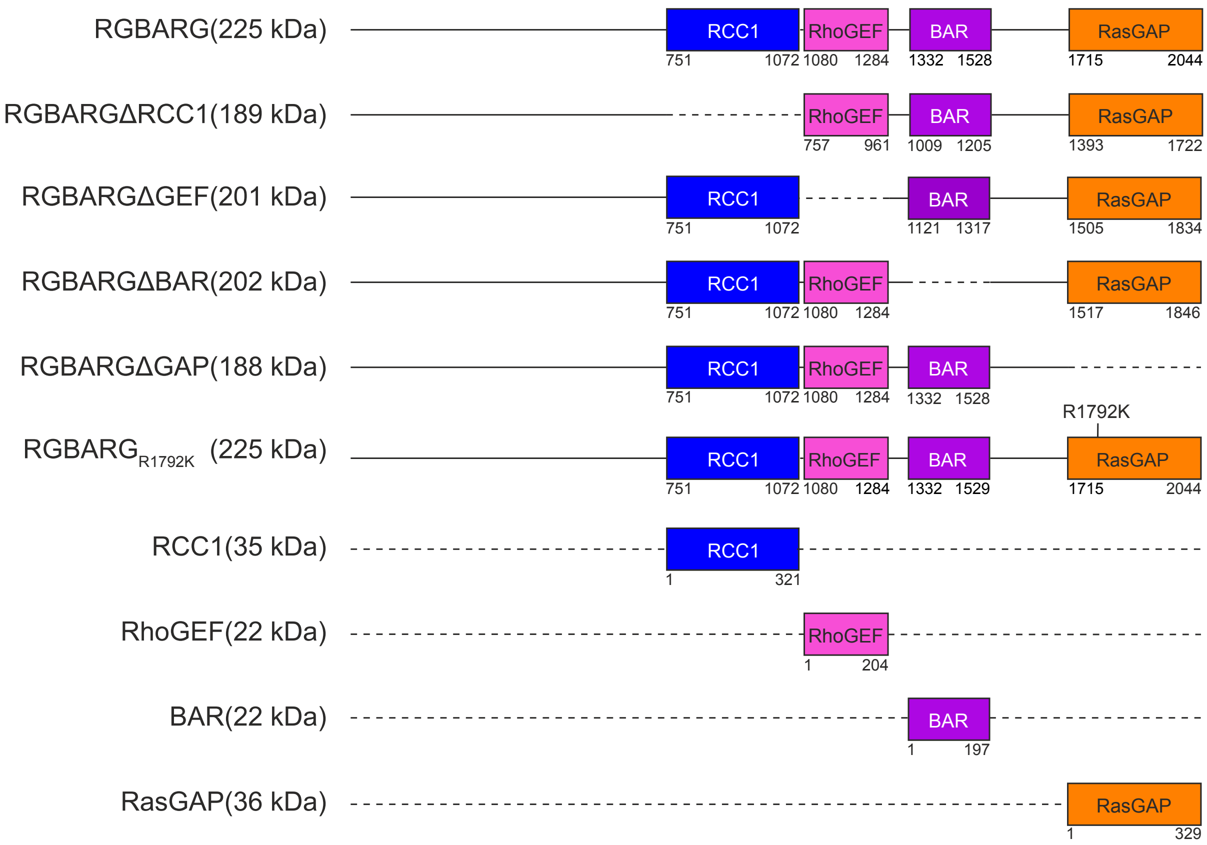

Figure S3: Schematic of the RGBARG truncation and point mutants used in this study, related to Figures 4 and 5

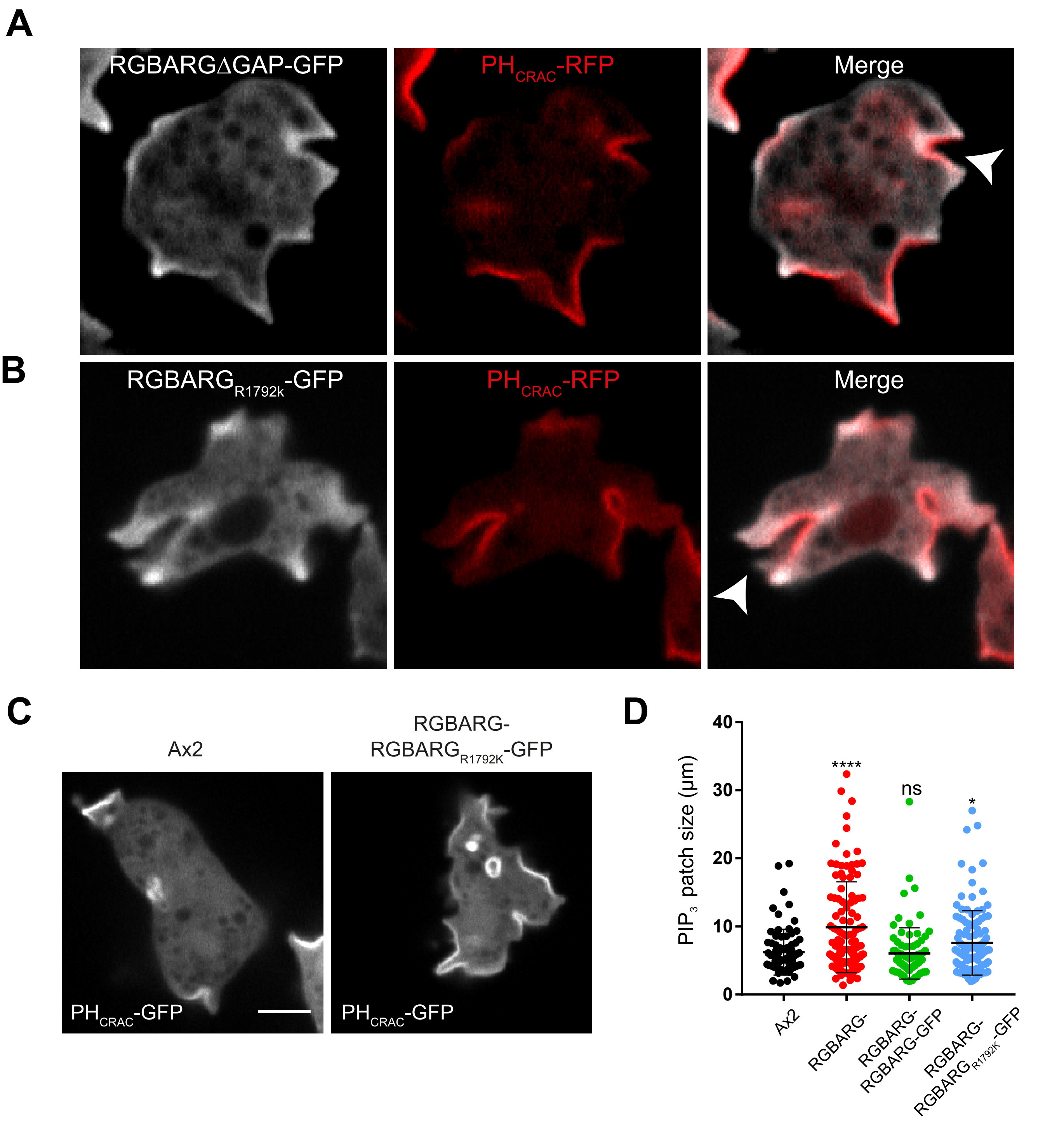

Figure S4: RGBARG$_{R1792K}$-GFP rescues RGBARG localisation, but not PIP$_3$ signalling, related to Figure 4. (A) Co-expression of RGBARGΔGAP-GFP and PH$_{CRAC}$-RFP in (Ax2) RGBARG- cells indicating that the RasGAP domain helps excluded RGBARG-GFP from PIP$_3$ rich regions of the cell. (B) The inactive GAP mutant R1792K localizes normally and is excluded from the base of protruding cups (arrowhead). (C) PH$_{CRAC}$-GFP localisation in Ax2 and RGBARG- cells expressing RGBARG$_{R1792K}$-GFP, demonstrating that PIP$_3$ dynamics are not rescued by this construct. (D) Quantification of PH$_{CRAC}$-GFP patch size.

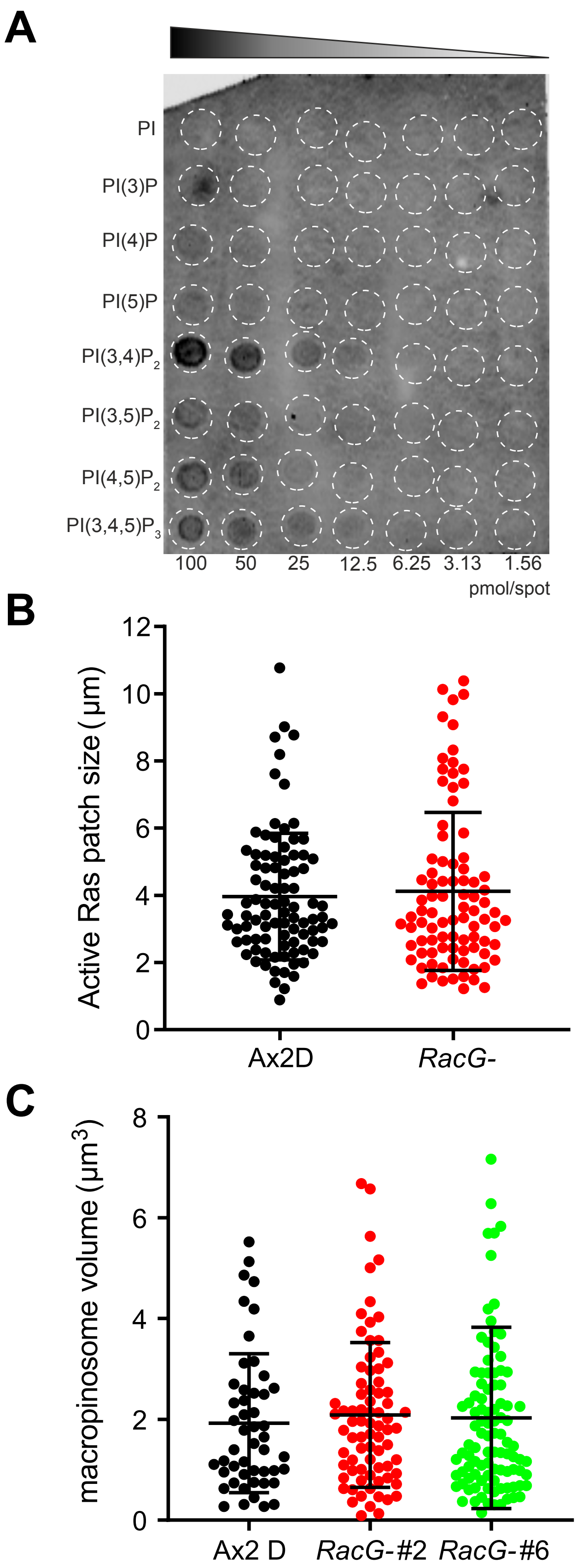

Figure S5: BAR domain lipid binding specificity, and macropinosome formation in RacG mutants, related to Figure 5. (A) PIP array analysis of BAR-GFP binding showing a moderate preference for $PI(3,4)P_2$ in this assay. (B) Active Ras signaling during macropinosome formation in RacG- cells and their parental cell line Ax2D. Patch size was quantified from single confocal planes of cells expressing RBD-GFP. (C) Volume of macropinosomes formed by RacG- and control cells, measured by imaging cells taking up FITC dextran. Two independent RacG- clones were analysed over 3 independent experiments.
